# Transcriptome Analysis of *Cryphonectria parasitica* Infected With Cryphonectria hypovirus 1 (CHV1) Reveals Distinct Genes Related to Fungal Metabolites, Virulence, Antiviral RNA-Silencing, and Their Regulation

**DOI:** 10.3389/fmicb.2020.01711

**Published:** 2020-07-17

**Authors:** Jeesun Chun, Yo-Han Ko, Dae-Hyuk Kim

**Affiliations:** ^1^Institute for Molecular Biology and Genetics, Jeonbuk National University, Jeonju, South Korea; ^2^Department of Bioactive Material Sciences, Jeonbuk National University, Jeonju, South Korea; ^3^Department of Molecular Biology, Jeonbuk National University, Jeonju, South Korea

**Keywords:** hypovirulence, RNA-Seq, transcriptomic analysis, *Cryphonectria parasitica*, Cryphonectria hypovirus

## Abstract

Comprehensive transcriptome analysis was conducted to elucidate the molecular basis of the interaction between chestnut blight fungus, *Cryphonectria parasitica*, and single-stranded RNA (ssRNA) mycovirus Cryphonectria hypovirus 1 (CHV1), using RNA-sequencing (RNA-seq). A total of 1,023 differentially expressed genes (DEGs) were affected by CHV1 infection, of which 753 DEGs were upregulated and 270 DEGs were downregulated. Significant correlations in qRT-PCR analysis of 20 randomly selected DEGs and agreement with previously characterized marker genes validated our RNA-seq analysis as representing global transcriptional profiling of virus-free and -infected isogenic strains of *C. parasitica*. Gene Ontology (GO) analysis of DEGs indicated that “cellular aromatic compound metabolic process” and “transport” were the two most enriched components in the “biological process.” In addition, “cytoplasm” was the most enriched term in the “cellular component” and “nucleotide binding” and “cation binding” were the two most enriched terms in the “molecular function” category. These results suggested that altered expression of genes encoding numerous intracellular proteins due to hypoviral infection resulted in changes in specific metabolic processes as well as transport processes. Kyoto Encyclopedia of Genes and Genomes function analysis demonstrated that pathways for “biosynthesis of other secondary metabolites,” “amino acid metabolism,” “carbohydrate metabolism,” and “translation” were enriched among the DEGs in *C. parasitica*. These results demonstrate that hypoviral infection resulted in massive but specific changes in primary and secondary metabolism, of which antiviral fungal metabolites were highly induced. The results of this study provide further insights into the mechanism of fungal gene regulation by CHV1 at the transcriptome level.

## Introduction

The *Cryphonectria parasitica*–Cryphonectria hypovirus 1 (CHV1) interaction is a successful natural biocontrol system mediated by a mycovirus. Hypoviral infection reduces the pathogenic potential of its host fungus, a phenomenon referred to as hypovirulence, with associated symptoms that include reduced asexual sporulation, sexual reproduction, and pigmentation in *C. parasitica* (Van Alfen et al., 1975; Nuss, 1992). Among the four established species of CHV1, 2, 3, and 4, CHV1 is the type species and the species on which most studies have been conducted, and shows all of the typical symptoms of hypoviral infection (Hillman and Suzuki, 2004; Nuss, 2005). Thus, infection of *C. parasitica* with CHV1 prevented a devastating chestnut blight epidemic in the European chestnut forests.

The interaction between fungus and virus involves the modulation of gene expression. The interaction between *C. parasitica* and CHV1 has been studied intensively at the molecular and cellular levels, aiming to identify genes involved in symptom development and the novel defense mechanism of fungi in response to hypoviral infection. The availability of full-length genome sequences of both *C. parasitica* and hypoviruses and relatively easy genetic manipulation of *C. parasitica* and CHV1 via gene replacement, stable heterokaryon formation, and infectious cDNA copy of CHV1 has made *C. parasitica*-CHV1 a model system for the study of fungus–virus interactions (Shapira et al., 1991; Choi and Nuss, 1992; Kim et al., 1995; Smart et al., 1999; Hillman and Suzuki, 2004; Nuss et al., 2010; Ko et al., 2016). Differential mRNA display (Chen et al., 1996; Kang et al., 2000) and cDNA microarray analysis representing approximately 2,200 unique genes (Allen et al., 2003) were conducted in *C. parasitica* to determine genes involved in key regulatory pathways affected by hypoviral infection. However, differential mRNA display requires considerable additional effort to determine the identities of differentially expressed genes (DEGs) and microarrays can provide information only on given sequences (Allen et al., 2003). By contrast, using high-throughput next-generation sequencing (NGS) methods, genome-wide transcript profiling can be carried out by directly sequencing the mRNA in a sample. Therefore, RNA sequencing (RNA-seq) is considered the most powerful tool for transcriptomic analysis. Although recent study using RNA-seq found 2,717 DEGs (Li et al., 2018), no further transcriptomic analysis of virus-infected and virus-free strains has been reported.

In this study, we conducted transcriptomic analysis using RNA-seq and compared transcript profiles between the hypovirus-infected and wild-type strains. Based on transcriptome analysis, a global view of transcriptional expression changes involved in the response of *C. parasitica* to CHV1 can be obtained, which will help elucidate the molecular interactions between hypoviruses and fungi.

## Results and Discussion

### RNA-Seq Dataset Summary

To identify transcripts that were differentially expressed in response to hypovirus CHV1 infection, we extracted RNA from the hypovirus CHV1-infected UEP1 strain and the virus-free isogenic EP155/2 strain, which were grown for 5 days on a cellophane overlay atop a potato dextrose agar plate supplemented with methionine and biotin (PDAmb), and then subjected to RNA-seq. A total of six libraries, including three biological repeats of each of the UEP1 and EP155/2 strains, were sequenced and the qualified reads were aligned using HISAT2. All the sequences were deposited to the sequence read archive (SRA) of the NCBI under the project accession number PRJNA588887. As a result, 61,826,456 total high-quality paired-end reads were generated using the Illumina Hiseq2000 sequencing platform, of which 59,289,126 reads, ranging from 94.69 to 99.26% of the high quality reads for each library, were successfully mapped to the reference genome of *Cryphonectria parasitica*^1^ predicting a total of 11,609 gene transcripts (Table 1). A total of 11,431 gene transcripts of the reference genome were represented in these mapped reads.

**TABLE 1:** Descriptive statistics of sequencing data.

**Sample**	**Biological replicates**	**Total high-quality reads generated**	**Total reads mapped to reference**	**Mapping rate (%)**
EP155/2	1	11,597,418	11,461,545	98.83
	2	11,036,016	10,888,630	98.66
	3	8,253,807	8,192645	99.26
UEP1	1	11,850,461	11,262,703	95.04
	2	10,687,981	10,120,801	94.69
	3	8,400,773	8,037,138	95.67

### Transcriptional Profile of *Cryphonectria parasitica* in Response to Hypovirus CHV1 Infection

**FIGURE 1:**
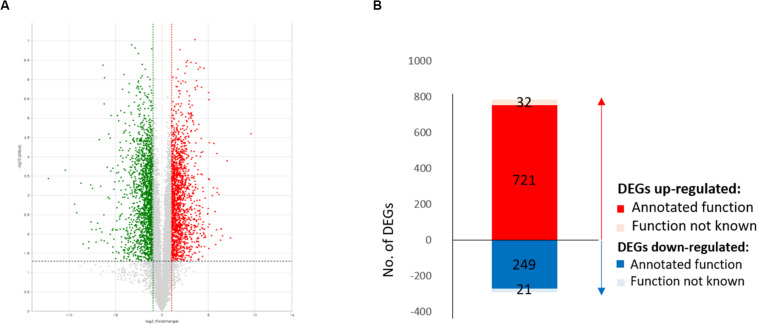
Differentially expressed gene (DEG) profiles of wild-type EP155/2 and hypovirus-infected UEP1. **(A)** Volcano plot showing the means (*x*-axis) and log_2_ ratios (*y*-axis) of genes sequenced in EP155/2 and UEP1. Red and green dots denote up- and downregulated genes, respectively, and black dots indicate genes with no significant expression. **(B)** Total number of DEGs (fold change > | 2|, FDR ≤ 0.01); up- and down-regulated DEGs are shown with red and blue lettering, respectively.

For functional annotation of genes, all 11,431 assembled sequences were applied to BLASTX searches against the NCBI non-redundant database as well as the GO and Kyoto Encyclopedia of Genes and Genomes (KEGG) databases, and a total of 10,033 unigenes with *E*-value of less than 1e-10 were functionally annotated. Based on pairwise comparison of counts for each transcript, a total of 1,023 DEGs (Supplementary Table S1), comprised of 753 upregulated and 270 downregulated genes, were identified with absolute values of log base 2 fold change (FC) of 1 or higher and *p*-values adjusted using a false discovery rate (FDR) of 0.01 in the transcriptomic comparison (Figure 1). Among the 753 upregulated and 270 downregulated genes, 721 and 249 were functionally annotated as upregulated and downregulated DEGs, respectively.

### Validation of DEGs Using qRT-PCR Analysis

**FIGURE 2:**
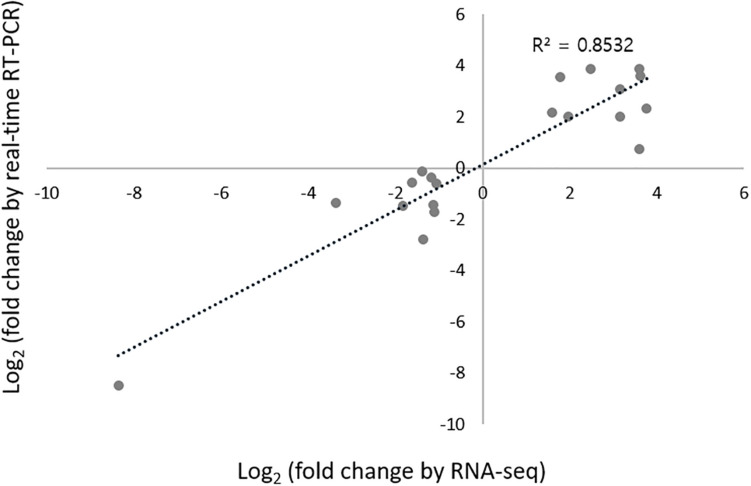
Validation of the expression of 20 randomly selected DEGs using RNA-seq and qRT-PCR analysis. Log_2_FC is calculated from the mean of three samples. The coefficient of determination (*R*^2^) is displayed.

To validate the RNA-seq data, quantitative real-time RT-PCR (qRT-PCR) was conducted for 20 randomly selected DEGs. Among these 20 DEGs, 10 were upregulated genes, including MFS monocarboxylate transporter, kelch domain-containing protein, hypothetical dynein assembly factor, putative arylesterase monooxygenase protein, *andM*, putative catalase, putative chitin synthase, and three with unknown functions. Ten were downregulated genes, including acid proteinase, putative GPI-anchored protein, putative acid phosphatase, phosphoesterase, putative erythrocyte band seven integral membrane protein, putative laccase2, cryparin, *S*-adenosylmethionine-dependent methyltransferase-like protein, ferric-chelate reductase, and one uncharacterized gene. To confirm the consistency of gene expression between RNA-seq and qRT-PCR results, we measured the correlation coefficient between logarithm-transformed RT-PCR expression values and qRT-PCR 2^–ΔΔ*CT*^ values. As the Shapiro–Wilk test revealed the normality of data at *p* < 0.05 (Royston, 1993), Pearson’s correlation analyses were performed. The overall Pearson’s correlation coefficient *R* was 0.8532 (*p* < 0.01), indicating that the correlation between RNA-seq and qRT-PCR results was highly significant (Figure 2), and thereby confirming the reliability of our RNA-seq platform.

### Validation of DEGs Through Comparison With Previously Characterized Genes

To verify our RNA-seq results, we screened a set of our DEGs for which expression had already been characterized and compared our findings with the published results. Table 2 shows the results of comparison between our current RNA-seq results for nine representative hypovirus-affected genes of *C. parasitica* and their characterizations in 11 published articles.

**TABLE 2:** Comparison of transcriptome responses to hypovirus and published data.

**JGI gene ID**	**Gene name**	**Expression pattern**	**Published data**		
			**Culture condition (strain/growth condition)**	**Technique**	**References**
104198	Cryparin	↓	UEP1/liquid	RNA slot-blotting	(↓) Zhang et al. (1994)
			UEP1/plate	RT-PCR	(↓) So and Kim (2017)
47319	Cutinase	↓	EP713/liquid	Northern blotting and cutinase assay	(↓) Varley et al. (1992)
97810	*Cppk1*	↑	UEP1/plate	Northern blotting	(↑) Kim et al. (2002)
79817	*Cpmk1*	↓	UEP1/plate	Western blotting	(↓) Park et al. (2004)
276559	*dcl2*	↑	EP713/plate	RT-PCR	(↑) Sun et al. (2009)
292762	*agl2*	↑	EP713/plate	RT-PCR	(↑) Sun et al. (2009)
345317	HSP70	↑	UEP1/plate	2-DE	(↑) Kim et al. (2012)
38462	*epn1*	↑	EP713/liquid	Northern blotting	(–) Choi et al. (1992)
			EP713/liquid	Microarray	(↓) Allen et al. (2003)
			EP713/liquid	iTRAQ based proteome	(–) Wang et al. (2016)
69386	*lac1*	↑	EP713/liquid	Microarray	(–) Allen et al. (2003)
			EP713/liquid	Northern blotting	(↓) Choi et al. (1992)
			EP713/liquid	iTRAQ based proteome	(↓) Wang et al. (2016)
			UEP1/plate	Laccase assay	(↓) Rigling and Van Alfen (1993)

*Arrows denote whether expression was upregulated (↑) or downregulated (↓). A minus sign (−) denotes no change in expression.*

#### Gene Known as a Morphological and Biochemical Marker

Hydrophobin cryparin, the most abundant fungal cell wall-associated hydrophobic protein, acts as a pathogenicity factor by facilitating the eruption of fruiting bodies (Kazmierczak et al., 2005). The cryparin gene *crp1* was highly expressed throughout the culture period, but transcriptionally downregulated with CHV1 infection (Zhang et al., 1994). The current RNA-seq data indicated that the cryparin gene was significantly downregulated upon CHV1 infection, in good agreement with the previously reported expression profile (So and Kim, 2017).

#### Gene With Enzymatic Activity

One of the infection-related proteins, cutinase, plays an essential role in degrading the plant cuticle and penetrating into the plant host (Varley et al., 1992). Previous evidence showed that expression of this gene was suppressed by the presence of hypovirus (Varley et al., 1992). In this study, we showed that cutinase gene expression was also downregulated due to hypovirus CHV1.

#### Genes Involved in Signal Transduction Pathways

Numerous studies have been conducted to reveal the roles of genes in signal transduction pathways related to fungal gene regulation by hypovirus CHV1. Although further investigation of the DEGs identified in signal transduction pathways is required, genes involved in signal transduction pathways for colonial morphogenesis and stress responses, such as *Cppk1* and *Cpmk1*, were identified. Upregulation of the *Cppk1* gene, which encodes a novel Ser/Thr protein kinase and results in microcolony formation with a loss-of-function mutation, was observed during hypoviral infection (Kim et al., 2002). RNA-seq and Northern blot analysis results were in agreement regarding *Cppk1* gene expression during hypoviral infection. In addition, *Cpmk1*, a mitogen-activated protein kinase (MAPK) homolog of the yeast high-osmolarity glycerol (HOG) MAPK gene *hog1*, was negatively regulated during hypoviral infection in our RNA-seq results, consistent with previous findings (Park et al., 2004). These findings suggest that hypoviral infection specifically affects several cellular signaling processes.

#### Genes Involved in Antiviral RNA Silencing

Posttranscriptional RNA interference-mediated antiviral defense is associated with two major factors, dicer-like nuclease (*dcl*) and Argonaut-like proteins (*agl*), which are involved in targeted degradation of viral RNA (Sun et al., 2009). A specific dicer gene, *dcl2*, was required for RNA silencing in response to viral infection and for induction of viral RNA recombination. In addition, a specific Argonaut-like protein encoded by *agl2* was required for induction of *dcl2* expression. These results show that the viral infection promoted a large increase in *dcl2* expression and a modest increase in *agl2* expression. Our RNA-seq analysis revealed that genes encoding posttranscriptional gene silencing-related proteins, including *dcl2* and *agl2*, were differentially expressed. Expression of *dcl2* and *agl2* was increased by 2-fold and 1.7-fold, respectively. The expression of these genes encoding antiviral defense proteins showed similar patterns with those in previously reported transcript accumulation profiles, suggesting active regulation of the RNA silencing response in *C. parasitica*.

#### Genes Involved in the Stress Response

The heat shock protein 70 (HSP70) family is highly conserved across species and its members play an important role in managing cells under stressful conditions. Some, but not all, HSP70 proteins are encoded by stress-responsive and hypovirus-induced genes (Kim et al., 2012). Genes corresponding to stress-responsive and hypovirus-induced HSP70 proteins were specifically upregulated with hypoviral infection in our RNA-seq analysis, which is consistent with previous results. In addition, glutathione *S*-transferase, which is known to play a significant role in defending the host against oxidative stress (Breitenbach et al., 2015), was upregulated.

#### Gene Involved in Viral Replication

We found an interesting gene correlated with viral gene expression, with a significantly upregulated DEG identified as a putative zinc knuckle domain-containing protein, which is similar to the ATP-dependent RNA helicase *glh-4* of *Caenorhabditis elegans*. The zinc knuckle domain, which is also found in retroviral gag proteins (De Guzman et al., 1998), is important for protein–protein interactions (Vo et al., 2001) and is involved in nucleotide processing, including chaperoning, splicing, transcriptional activation, and termination (Darlix et al., 2002). Based on a GO analysis, this DEG was identified as “viral genome replication” (GO:0019079) under the term “multi-organism process” (GO:0051704). This result suggests that our RNA-seq analysis was sufficiently thorough to identify a host factor involved in viral replication, and also suggests that the current analysis is highly reliable.

#### Comprehensive Comparison of Technologies

Notably, the current RNA-seq analysis exhibited some inconsistency with published results for the expression level of a gene encoding the aspartic protease endothiapepsin, *epn-1*. Upon CHV1 infection, the production and secretion of the endothiapepsin protein showed no change in a previous study (Choi et al., 1993). However, a decreased level of the *epn-1* transcript was observed in microarray analysis (Allen et al., 2003) and secretome analysis revealed the endothiapepsin precursor as a downregulated secreted protein with no change in the corresponding transcript level (Wang et al., 2016). Our RNA-seq analysis showed that *epn-1* was an upregulated DEG. Transcriptional changes in a laccase, polyphenol oxidase, is another example. Three different laccase types, namely extracellular, intracellular, and inducible, are present in *C. parasitica* and all are affected by hypoviral infection (Kim et al., 1995). Microarray analysis showed that hypoviral infection did not affect the transcript level of the extracellular laccase, *lac1* (Allen et al., 2003; Wang et al., 2016), which has been shown to be downregulated with hypoviral infection previously (Choi et al., 1992; Rigling and Van Alfen, 1993; Wang et al., 2016). Our current results revealed that the *lac1* transcript was slightly but significantly upregulated. By contrast, *lac2* was downregulated and *lac3* showed no change without further induction, such as through supplementation of tannic acid, in accordance with previous studies (Chung et al., 2008; Kim et al., 2010). Moreover, the regulation of expression of fungal enzymes such as endothiapepsin and laccase is under multiple layers of regulatory mechanisms, including transcription, post-transcriptional modification, translation, and post-translational processing.

Several approaches have been used to characterize transcriptional changes in *C. parasitica* infected by hypoviruses based on RNA differential display, cDNA microarray, and RNA-seq analysis technologies combined with various culture conditions and strains used as isogenic pairs. Kang et al. (2000) revealed that approximately 10–20% of amplified transcripts, ranging from 768 to 1,920 bands, appeared to be modulated in the presence of viruses based on ordered differential display using RT-PCR (ODD-PCR) after a 7-day culture period on PDAmb plates. In addition, more than 400 PCR products were identified as DEGs related to hypoviral infection using conventional differential display protocols with a 6-day culture period on PDA overlain with cellophane (Chen et al., 1996). In microarray analyses of 2,200 expressed sequence tags, a total of 295 DEGs were identified with 6-day culture on PDA with cellophane, of which 132 genes were upregulated and 163 genes were downregulated (Allen et al., 2003). Although no further description was provided, a recent report based on RNA-seq analysis identified 2,717 DEGs with 7-day culture on PDA, of which 1,207 and 1,510 genes were upregulated and downregulated, respectively (Li et al., 2018).

Our RNA-seq analysis indicated that approximately 10.1% (1,023/10,033) of gene analyzed were DEGs, similar to the ratios observed in previous studies using RNA differential display, which ranged from 768 to 1,920 DEGs, and microarray analysis (13.4%), but smaller than the result from a recent RNA-seq study (2,717 DEGs). Interestingly, the ratio of up- to down-regulation (7.4:2.6), indicating the direction of differential expression, differed from those of previous studies in magnitude and direction; i.e., ratios of (4:5), (4.5:5.5), and (4.4:5.6) were obtained in RNA differential display analysis using ODD-PCR, microarray analysis, and RNA-seq analysis, respectively. Similar to our results, a ratio of 7:3 was obtained previously using RNA differential display (Chen et al., 1996). Considering that our RNA-seq results showed strong correlations with those of the validation methods, including significant correlations with qRT-PCR results for randomly selected DEGs and concordance with previously characterized CHV1-specific fungal genes, our RNA-seq results represent transcriptional changes in the host fungus due to our experimental condition of CHV1 infection. The discrepancy between published observations and the results described here can be attributed to differences in various factors that may alter gene expression, including differences in culture type (liquid vs solid), incubation time, and growth medium. Intrinsic differences may also exist between the isogenic pairs of virus-free and infected strains compared (i.e., EP155/2 vs UEP1, EP155 vs EP713), as UEP1 was generated by transferring hypovirus RNA from EP113 (ATCC 38771), a hypovirus-containing hypovirulent strain from Europe, to EP155 via the *met1* auxotroph EP2001 (ATCC 60589) strain via anastomosis (Powell and Van Alfen, 1987), whereas EP 713 (ATCC 52571; Anagnostakis and Day, 1979; Hillman et al., 1990) was obtained directly by transferring the hypovirus from EP113 to strain EP155. Therefore, unexpected differences between the pairs may have arisen through cytoplasmic inheritance from the hypovirus-donating strains, in addition to those arising from the hypovirus itself. Moreover, during the long history of a laboratory standard strain, the accumulation of mutations or epigenetic changes may occur unnoticed. In addition, although the RNA-seq platform appears to offer an immense advantage of detecting a larger number of genes affected by hypoviral infection, RNA-seq analysis appears to depend strongly not only on experimental conditions but also on the parameters of the analytical tools used to identify DEGs for the following reasons. First, our RNA-seq results showed a smaller number of DEGs (1,023 vs 2,717) and a different regulatory direction (greater proportion of upregulated vs downregulated gene) compared to previous RNA-seq analyses. Secondly, only 402 DEGs were observed in both studies and for those DEGs, no correlations (Pearson’s correlation coefficient *R* of 0.0001) was observed between the two RNA-seq analyses (Supplementary Figure S1). These results reinforce the importance of validation. Compared to previous studies that have used differential hybridization (Powell and Van Alfen, 1987), differential display, and microarray analyses, RNA-seq detected a significantly larger number of DEGs. In addition, comparative analyses of RNA-seq results and recently published proteome data (Kim et al., 2012; Wang et al., 2016) in response to hypoviral infection revealed no correlations (correlation coefficient *R* less than 0.001), which was consistent with previous studies showing a poor correlation between mRNA and protein abundances (Maier et al., 2009). Thus, the larger independent dataset used here is beneficial for obtaining deeper insights into the molecular interactions between hypoviruses and their hosts. Notably, the culture conditions and analytical pipelines used with NGS data have strong impacts on the results, and therefore further validation is required for genes of interest.

#### Unique Genes Not Identified by Other Technologies

Because our RNA-seq analysis showed that only a small portion (402 out of 1,023) of DEGs were common DEGs, with no correlation to previous RNA-seq data, all DEGs, including common and unique DEGs, were further characterized according to their putative functions in GO and KEGG analyses. In addition, the 10 most up- and down-regulated DEGs among common and unique DEGs are shown in a new representative table (Table 3).

**TABLE 3:** List of unique and common DEGs showing the highest expression compared to a previous RNA-seq study (Li et al., 2018).

	**Unique genes**		**Common genes**		
	**Gene ID**	**Function description**	**Gene ID**	**Function description**	**Previous RNA-seq analysis**
Up-regulated DEGs	15980	Six-bladed beta-propeller	333952	Oxidoreductase	↑
	253279	Hypothetical protein	277466	Putative NAD dependent epimerase/dehydratase	↓
	220966	Pyoverdine biosynthesis protein	358238	Unknown function	↓
	37244	Putative amino acid permease	11941	Putative aristolochene synthase protein	↑
	255304	Putative alpha/beta-hydrolase protein	67771	Lovastatin nonaketide synthase	↓
	248043	Sterol 24-*C*-methyltransferase	251667	Putative major facilitator superfamily transporter	↓
	296388	Alkanesulfonate monooxygenase	67772	Putative polyketide synthase	↓
	343514	Putative cytochrome p450	260972	Putative globin-like protein	↓
	358138	Cupin 2	77536	Peptide transport protein PTR2	↓
	295721	Putative NADH:flavin oxidoreductase/NADH oxidase family protein	356776	Unknown function	↑
Down-regulated DEGs	354114	Ferric reductase like transmembrane component	67777	Putative 1,3-beta-glucanosyltransferase gel3	↑
	347713	Baeyer-Villiger monooxygenase	343827	Putative formyl transferase domain-containing protein	↑
	323704	Putative GNAT family	351545	Hexose transporter	↑
	340992	Putative linoleate diol synthase	292843	Putative polyketide synthase	↓
	358516	Putative pathogen-related protein	85578	Unknown function	↑
	357240	Hypothetical protein	357113	Unknown function	↓
	322863	Unknown function	80584	Nonribosomal peptide synthetase-like protein	↑
	335052	Unknown function	356961	Hypothetical protein	↓
	323047	Noranthrone monooxygenase	356957	Putative HC-toxin efflux carrier TOXA	↓
	323049	Beta-lactamase-like protein 2	279251	Uncharacterized protein	↓

*Arrows denote whether expression was upregulated (↑) or downregulated (↓).*

### GO Enrichment Analysis

**FIGURE 3:**
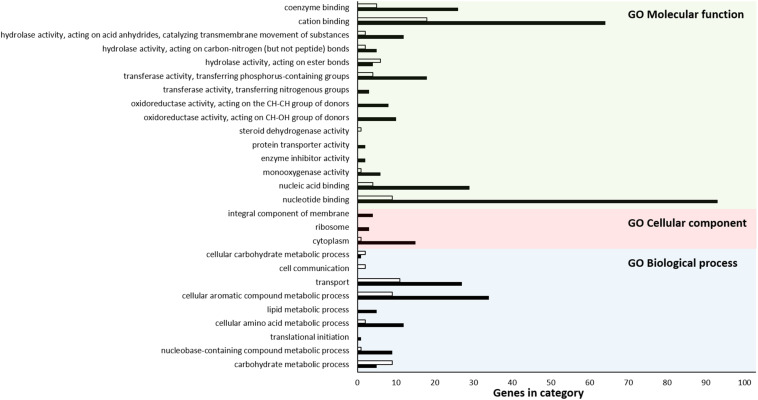
GO functional annotation histogram of DEGs. The GO term for each bar is indicated under the *x*-axis. The *y*-axis represents GO terms. Black and white colors represent the numbers of up- and down-regulated DEGs, respectively. The *x*-axis represents the number of genes showing differential expression. Three classifications based on the GO annotation categories of biological process, cellular component, and molecular function are denoted at the top of the bars as BP, CC, and MF, respectively.

Of 753 upregulated and 270 downregulated genes, 451 and 165, respectively, were classified into one or more GO terms based on sequence homology. In the biological process category, the GO terms “cellular aromatic compound metabolic process” (GO:0008152) (34 upregulated and 9 downregulated genes) and “transport” (GO:0009987) (27 upregulated and 11 downregulated genes) were the most highly enriched. The GO term “cytoplasm” (GO:0044464) (15 upregulated and one downregulated gene) was the most highly represented group in the cellular component category. For the molecular function category, “nucleotide binding” (GO:0003824) (93 upregulated and nine downregulated genes), and “cation binding” (GO:0005488) (64 upregulated and 18 downregulated genes) were the dominant groups (Figure 3). More upregulated DEGs than downregulated DEGs were present in almost all categories. On the other hand, several GO terms such as “microtubule-based process” (GO:0007017), “ubiquitin-like protein transferase activity” (GO:0019787), “protein dimerization activity” (GO:0046983), and “iron-sulfur cluster binding” (GO:0051536) were not significantly enriched, suggesting that only specific biological processes were affected by hypoviral infection. Thus, based on GO term analysis, global transcriptomic changes due to hypoviral infection were attributed to the modulation of gene expression via specific signaling pathways and transcription factors (TFs), which resulted in changes to metabolic processes carried out by cytoplasmic proteins and transporters.

### Pathway Enrichment Analysis

**FIGURE 4:**
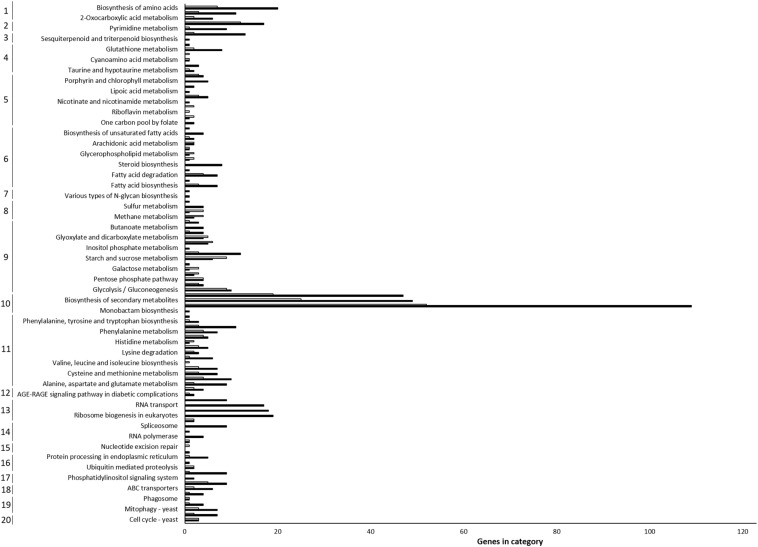
KEGG pathway enrichment analysis. Significant KEGG pathways among up-regulated (black) and down-regulated (white) DEGs are shown. DEGs were assigned to 20 categories, indicated by numbers on the left according to the biological pathway: (1) overview; (2) nucleotide metabolism; (3) metabolism of terpenoids and polyketides; (4) metabolism of other amino acids; (5) metabolism of cofactors and vitamins; (6) lipid metabolism; (7) glycan biosynthesis and metabolism; (8) energy metabolism; (9) carbohydrate metabolism; (10) biosynthesis of other secondary metabolites; (11) amino acid metabolism; (12) endocrine and metabolic diseases; (13) translation; (14) transcription; (15) replication and repair; (16) folding, sorting and degradation; (17) signal transduction; (18) membrane transport; (19) transport and catabolism; and (20) cell growth and death.

To evaluate the pathways that play important roles in fungus–hypovirus interactions, the DEGs were further assigned to 20 categories using KEGG pathway analysis. Although variations among the number of DEGs were found, the main pathways of “biosynthesis of other secondary metabolites” (303 DEGs), “amino acid metabolism” (113 DEGs), “carbohydrate metabolism” (104 DEGs), and “translation” (67 DEGs) were significantly represented (Figure 4), indicating that hypoviral infection leads to dramatic changes in specific metabolites and corresponding proteins in the fungal host. Within the KEGG category “biosynthesis of other secondary metabolites,” the three main sub-classifications were “metabolic pathway,” “biosynthesis of secondary metabolites,” and “biosynthesis of antibiotics.” These results suggested that most DEGs in primary and secondary metabolic pathways resulted from their involvement in hypovirus-specific growth and development processes of the fungal host. In previous studies based on metabolomic and microarray analyses, hypoviral infection resulted in a major impact on the primary metabolism of the fungal host by altering the synthesis of a variety of amino acids, carbohydrates, and lipids metabolites, including methionine, alanine, tyrosine, glucose, galactose, ether lipids, and other lipids (Allen et al., 2003; Dawe et al., 2009), which was reflected in the present results. Thus, KEGG metabolic pathway analysis in this study successfully confirmed the enrichment of pathways reported in previous metabolic results, and provided more detailed descriptions of the corresponding transcripts.

#### Global Gene Expression of Secondary Metabolic Genes

Fungi produce diverse secondary metabolites upon viral infection (García-Pedrajas et al., 2019; Nerva et al., 2019), which indicates that controlling viral replication and fungal protection against viral infection leads to the synthesis of secondary metabolites. Aside from accumulating primary metabolites, *C. parasitica* showed changes in a vast majority of secondary metabolites, including alkaloids, terpenoids, and polyketides, which may act as a barrier defense against infecting viruses (Linnakoski et al., 2018). Therefore, in addition to our KEGG pathway analysis, which was adopted from that for *Magnaporthe oryzae*, we further analyzed the 303 DEGs assigned to the Joint Genome Institute reference KEGG pathways. Among these, 81 DEGs were mapped to diverse pathways under the sub-classification “biosynthesis of other secondary metabolites” and most DEGs were assigned to the pathway “isoquinoline alkaloid biosynthesis” (11 up and one downregulated gene) and “carotenoid biosynthesis” (10 up- and one downregulated gene). These results suggest that biosynthesis of isoquinoline alkaloid and carotenoid was strongly induced by hypoviral infection. In addition, 14 DEGs were identified in the sub-classification of “biosynthesis of alkaloids derived from terpenoid and polyketide.” Among these, ten were upregulated and four were downregulated.

To identify changes in polyketide biosynthesis, we analyzed our 1,023 DEGs using the PKS and NRPS clusters (see text footnote 1), and found that five DEGs belonged to PKS, two were NRPS, and one was a hybrid. Among these, five DEGs including three PKS and one each of the NRPS and hybrid groups were upregulated, whereas three were downregulated. The most upregulated DEG was non-reducing polyketide synthase, which is similar to the gene *andM* of *Penicillium patulum*, encoding fatty acid synthase subunit alpha for the biosynthesis of anditomin. The most downregulated DEG encoded a highly reducing polyketide synthase similar to the *azaB* gene of *Aspergillus niger*. These results suggest alteration of polyketide profiles within the cell following hypoviral infection.

This analysis showed that fungal bioactive agents reported to act as antiviral agents, such as indole alkaloids, non-ribosomal peptides, polyketides, NRPS-PKS hybrids, and terpenoids, were induced or altered during viral infection (Chen et al., 2012; Rincão et al., 2012).

#### Analysis of DEGs in Amino Acid Metabolism in Response to Hypovirus

Viruses have been shown to alter numerous primary processes in their host fungi. A total of 113 DEGs involved in amino acid metabolism were up- or downregulated upon hypoviral infection. As this fungus has no nitrogen fixation capability and the pathways to amino acid synthesis arise as branching points from key intermediates in central metabolic pathways, such as the tricarboxylic acid cycle (TCA), glycolysis, and pentose phosphate pathway, DEGs belonging to the pathways for synthesis of almost every amino acid are present in this fungus.

These 113 DEGs included 14 DEGs (10 up- and four downregulated) for “glycine, serine, and threonine metabolism,” which promotes the TCA cycle. In addition, 34 DEGs were represented for aromatic amino acid metabolism, among which 14, 11, and 9 DEGs were related to the metabolism of “tryptophan,” “phenylalanine,” and “tyrosine,” respectively. Four DEGs were mapped to the pathway of aromatic amino acid biosynthesis. Seven and five DEGs were involved in “lysine biosynthesis” and “lysine degradation,” respectively. A total of 11 DEGs were included in “alanine, aspartate, and glutamate metabolism,” which are derived from intermediates of the TCA cycle. For metabolism of branched-chain amino acids such as valine, leucine, and isoleucine, which are all derived from pyruvate, a total of 11 DEGs were identified. Among these 11, 10 DEGs (seven up- and three downregulated genes) were for “degradation” and only one downregulated DEG was reported for “biosynthesis,” suggesting a high rate of conversion of branched-chain amino acids such as valine, leucine, and isoleucine into acyl-CoA derivatives. Ten DEGs were involved in “cysteine and methionine metabolism,” representing sulfur-containing amino acids. Eight and six DEGs were involved in “arginine and proline metabolism” and “arginine biosynthesis,” respectively. Three DEGs were related to histidine, in pathways such as “histidine metabolism.”

#### Analysis of DEGs in Response to Hypovirus Related to Carbohydrate Metabolism

Carbohydrate-related pathways were also enriched. A total of 104 genes including those related to glycolysis/gluconeogenesis (10 up- and nine downregulated genes), starch and sucrose metabolism (six up- and nine downregulated genes), amino sugar and nucleotide sugar metabolism (12 up- and three downregulated genes), pyruvate metabolism (five up- and six downregulated genes), glyoxylate and dicarboxylate metabolism (four up- and five downregulated genes), the pentose phosphate pathway (four up- and four downregulated genes), citrate cycle (four up- and three downregulated genes), fructose and mannose metabolism (two up- and three downregulated genes), propanoate metabolism (four up- and one downregulated genes), butanoate metabolism (four upregulated genes), galactose metabolism (one up- and three downregulated genes), ascorbate and aldarate metabolism (one upregulated gene), and inositol phosphate metabolism (one upregulated gene) were identified. These genes were further assigned to carbohydrate-active enzyme (CAZy) families in relation to cell wall polysaccharide modifications using the CAZy database. Glycoside hydrolases (GH) (16 DEGs) and glycosyltransferase (GT) (16 DEGs) families were most prevalent among CAZymes. These results indicate that hypoviral infection regulates multiple aspects of carbohydrate metabolism involving not only hydrolysis, but also the formation of glycosidic bonds, resulting in changes to virulence and other associated phenotypic changes.

#### Genes Encoding Predicted Components of Transportation, Translation, and Signal Transduction Pathways

RNA-seq data indicated that several types of transporter activity genes were significantly up- or downregulated. Among these genes were 26 primary active transporters (22 up- and four downregulated genes) including ABC transporters and peptide transporters, and 54 electrochemical potential-driven transporters (41 up- and 13 downregulated genes) such as MFS transporters and nutrient porters, six transmembrane electron carriers (three up- and three downregulated genes) including ferric reductase, six channel/pore proteins (six upregulated genes) such as ion transporters for potassium and calcium, and two group translocators (two downregulated genes). Although further studies are required to determine the biological consequences of these changes in transcription, changes in specific transporters responsible for primary and secondary metabolites suggest that these specific transporters affect specific metabolite flows within the cell during viral infection. In addition, translation pathways were represented, including aminoacyl-tRNA biosynthesis, ribosome biogenesis in eukaryotes, ribosome, RNA transport, and mRNA surveillance pathway. These findings suggest that hypoviral infection resulted in dramatic changes in *de novo* protein synthesis, accounting for 5% of total protein content based on 2-D analysis of protein products (Powell and Van Alfen, 1987).

During molecular interactions between the fungus and hypovirus, host MAPKs are activated for fungal immune signaling. Among nine DEGs related to molecular transducer activity, high osmotic-related MAPK, HOG1, and serine/threonine kinases, which are involved in signal transduction pathways, were significantly expressed.

### Transcription Factor Genes

Genes encoding TFs were annotated based upon Pfam domain analysis, and these genes were highly represented in our analysis. In this study, 32 TFs in seven families were identified among 1,023 DEGs. The most highly represented classes were the fungus-specific Zn_2_-C_6_ binuclear cluster domain with 14 DEGs and the C_2_H_2_ zinc finger cluster with 11 DEGs. These TFs generally play critical roles in environmental regulatory responses (Chen and Zhu, 2004) and are associated with the production of secondary metabolites (Keller et al., 2005) as well as stress responses (Shelest, 2008). Although the specific target genes of the 32 annotated TFs are not yet known, their identification and examination of their regulatory mechanisms are interesting topics for future research.

These results of TF analysis strongly suggest that *C. parasitica* recognized CHV1 infection as a biotic stressor and underwent massive metabolic changes to maintain its fundamental biological processes. The dramatic changes caused by CHV1 infection were well represented in metabolic pathways relating to coping with CHV1 infection by inducing antiviral defense systems, including RNA silencing and biosynthesis of antibiotics. These direct cause-effect relationships were accompanied by diverse changes, resulting in hypovirulence and abnormal fungal development. Hypoviral infection affected not only secondary metabolites but also primary metabolites. Not all primary metabolites were affected, and instead a specific set of primary metabolites was affected by the presence of hypovirus. In addition, many examples of changes in specific types of secondary metabolites were observed. These results clearly indicated that the response of *C. parasitica* to hypoviral infection was specifically tailored to cause viral symptoms.

## Conclusion

In this study, transcriptome data provide comprehensive elucidation of fungal responses to hypoviral infection based on RNA-seq technology. A total of 1,023 DEGs were identified, of which 753 were upregulated and 270 were downregulated. Compared to the microarray and differential mRNA display platforms, the RNA-seq platform identified a larger number of DEGs upon hypoviral infection, and these DEGs were supported by through validation. In GO analysis, significant numbers of DEGs were classified into the biological process, cellular component, and molecular function terms. GO enrichment analysis suggested that global transcriptomic changes could be attributed to transcriptional regulation of specific sets of genes involved in metabolic processes carried out by cytoplasmic and membrane proteins. Pathway analysis indicated that transcriptional changes in response to hypoviral infection convey abundant information on the genes involved in various pathways, including the four major pathways of “biosynthesis of other secondary metabolites,” “amino acid metabolism,” “carbohydrate metabolism,” and “translation.” The RNA-seq results presented here provide information about global transcriptional changes caused by hypoviral infection, which include changes in not only primary but also secondary metabolites, as well as changes in the regulation of the corresponding genes via intracellular signaling and pathway-specific TFs. This study will help elucidate the molecular mechanisms underlying fungus–mycovirus interactions.

## Materials and Methods

### Fungal Strains and Growth Conditions

*Cryphonectria parasitica* wild-type strain EP155/2 (ATCC 38755) and its isogenic hypovirus-CHV1-containing strain UEP1 were grown on cellophane membranes overlaying potato dextrose agar (PDA, Difco Laboratories, United States) supplemented with methionine and biotin (PDAmb) in Petri dishes (90 mm diameter) under constant light (2,000 lx) at 25°C in a microbiological incubator (Zhang et al., 1993; Kim et al., 1995).

### RNA Extraction and RNA-Seq Data Processing

Three fungal agar plugs were inoculated onto cellophane membranes overlaying PDAmb medium and incubated at 25°C for 5 days. Mycelia from 20 cultured dishes of each strain were pooled, and biologically independent experiments were carried out in triplicate on three separate days. Harvested mycelium was quickly frozen and ground in liquid nitrogen. The powder was resuspended in 20 mL lysis buffer [200 mM Tris-HCl pH 7.5, 250 mM NaCl, 50 mM ethylenediaminetetraacetic acid (EDTA), 200 mM para-aminosalicylate sodium (PAS-Na), 20 mM triisopropyl naphthalene sulfonic acid (TNS)] per gram mycelium and an equal volume of water-saturated phenol, and total RNA was extracted through repeated rounds of purification with phenol:chloroform and lithium chloride precipitation. The extracted RNA was treated with RNase-free DNase I (Promega, Madison, WI, United Kingdom). RNA quantity and quality were determined using a Nanodrop spectrophotometer and Bioanalyzer. cDNA library pools from three biological repeats of each sample were prepared and assessed through sequencing on the Illumina HiSeq 2000 system, which generated 6.6 Gbp of 101-bp paired-end reads per sample. The resulting reads were trimmed based on quality of phred score of more than 20 and low-quality reads less than 25 bp were filtered using SolexaQA (Cox et al., 2010). The clean qualified data from each sample were mapped to the annotated gene transcripts of the reference *C. parasitica* genome (see text footnote 1) using Bowtie 2 (v2.1.0) software (Langmead and Salzberg, 2012). Read counts were normalized using the DESeq library (Anders and Huber, 2010) implemented in the software R package.

### RNA-Seq Data Analysis

The expression level of an assembled unique gene was calculated and normalized using the fragments per kilobase of exon per million (FPKM) method by dividing the number of fragments mapped to each gene by the size of its transcripts. FDR obtained by converting the statistical score to a *p*-value using a binomial distribution, was applied to rule out the possibility of false positives and identify the threshold *p*-value. Genes with *p*-values less than 0.01 were regarded as significantly expressed. FC analysis was performed to identify DEGs between each pair of samples. DEGs were identified based on two-fold changes in FPKM read counts for all pairwise comparisons. To assess the differential transcript levels in pairwise comparisons, thresholds of log_2_-fold changes in expression larger than 1 and smaller than −1 were taken into consideration as significantly up- and downregulated in this study, respectively. Only genes with FDR < 0.01 and log_2_| fold change| > 1 were reported as DEGs. BLASTX alignment using a cutoff *E*-value of 1e-10 was employed for functional annotation of DEGs. The NCBI non-redundant protein and KEGG pathway databases of the reference strain *Magnaporthe oryzae* were used to decide the functional annotation of unigenes with statistical significance based on a *p*-value less than 0.01. Blast2GO was used to sort GO terms into biological process, cellular component, and molecular function categories (counts ≥ 1).

### Validation of DEGs Through Quantitative Real-Time RT-PCR Analysis

Quantitative real-time RT-PCR analyses were carried out to validate the RNA-seq results for 20 selected DEGs. Total RNA was extracted again from each sample cultured for 5 days on PDAmb plates as described above, and first-strand cDNA was synthesized from 500 ng RNA using SuperScript IV reverse transcriptase (Invitrogen Corp., Carlsbad, CA, United States) with random primers. Real-time RT-PCR was performed using the Applied Biosystems 7500 system with SYBR premix Ex Taq II (TaKaRa, Tokyo, Japan). The reaction mixture consisted of 10 μL premix enzyme, 10 μM each primer, and 2 μL cDNA in a total volume of 20 μL. Primer sequences for 20 selected DEGs are shown in Supplementary Table S2. Cycling conditions were 30 s at 95°C for initial denaturation, followed by 30 cycles of 5 s at 95°C and 30 s at 60°C. Analyses were conducted for each transcript in three technical replicates, and at least three biological replicates were performed using independent RNA preparations for each sample. Transcript levels were normalized to the mRNA values of the internal control gene glyceraldehyde-3-phosphate dehydrogenase (GenBank No. P19089), and relative gene expression level was analyzed using the 2^–ΔΔ*CT*^ method (Livak and Schmittgen, 2001). The correlation between results from the two analysis platforms, RNA-seq and qRT-PCR, was analyzed by calculating the Pearson’s correlation coefficient, which had a *p*-value less than 0.01, using SPSS software (ver. 23.0, SPSS Inc., Chicago, IL, United States).

### Statistical Analysis

All qRT-PCR transcripts were analyzed via analysis of variance (ANOVA) using SPSS software (ver. 23.0, SPSS Inc., Chicago, IL, United States). The significance of all effects was determined using the Student-Newman-Keuls method at a significance level of *p* = 0.01.

## Data Availability Statement

All the sequences were deposited to the sequence read archive (SRA) of the NCBI under the project accession number PRJNA588887.

## Author Contributions

D-HK supervised the study and reviewed the manuscript. JC and Y-HK performed the experiments and analysis. D-HK and JC wrote the manuscript. All authors read and approved the final manuscript.

## Conflict of Interest

The authors declare that the research was conducted in the absence of any commercial or financial relationships that could be construed as a potential conflict of interest.

## Footnotes

1 http://genome.jgi-psf.org/Crypa2/Crypa2.home.html
