# Supplementary figures and images for: Transcriptome Analysis of Cryphonectria parasitica Infected With Cryphonectria hypovirus 1 (CHV1) Reveals Distinct Genes Related to Fungal Metabolites, Virulence, Antiviral RNA-Silencing, and Their Regulation
Source: Front Microbiol. 2020 Jul 17;11:1711. doi: 10.3389/fmicb.2020.01711 (PMC7379330; doi:10.3389/fmicb.2020.01711)

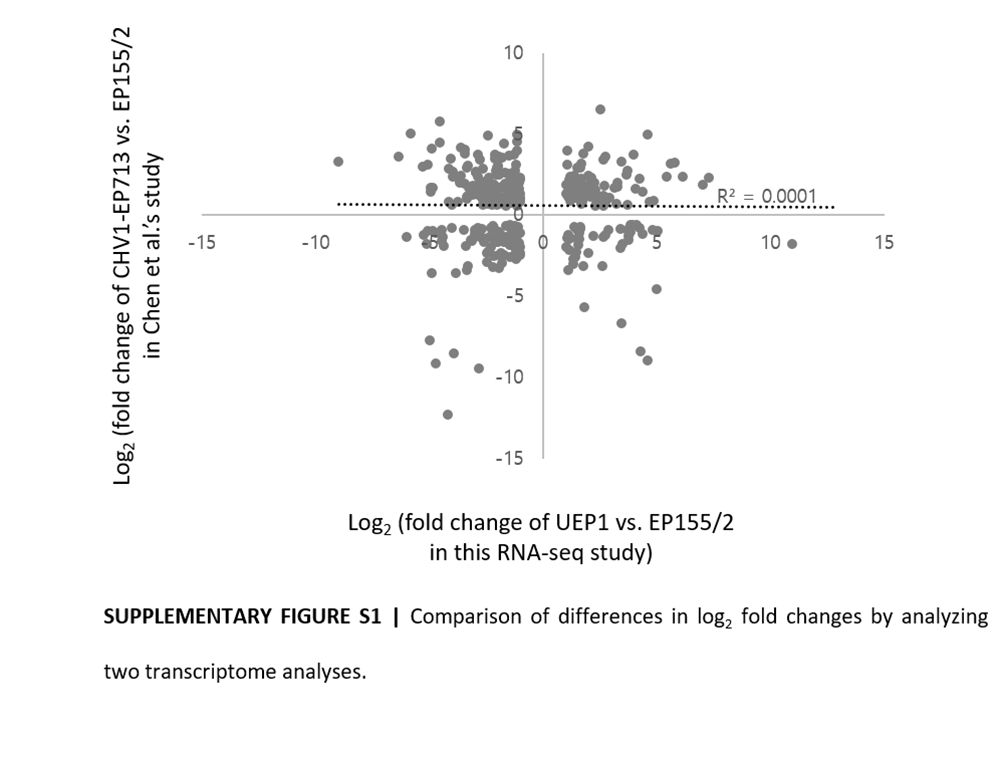

Supplement: Supplementary file 1 [file Image_1.TIF]
