# Supplementary material for: Transcriptome Analysis of Cryphonectria parasitica Infected With Cryphonectria hypovirus 1 (CHV1) Reveals Distinct Genes Related to Fungal Metabolites, Virulence, Antiviral RNA-Silencing, and Their Regulation
Source: Front Microbiol. 2020 Jul 17;11:1711. doi: 10.3389/fmicb.2020.01711 (PMC7379330; doi:10.3389/fmicb.2020.01711)
Supplement: Supplementary file 2 [file Table_1.DOCX]

**Table S1 |** Raw count of RNA-seq data

| **Raw count** | | | | | | |
| --- | --- | --- | --- | --- | --- | --- |
| **JGI ID** | **EP155/2**  **Repeat 1** | **EP155/2**  **Repeat 2** | **EP155/2**  **Repeat 3** | **UEP1**  **Repeat 1** | **UEP1**  **Repeat 2** | **UEP1**  **Repeat 3** |
| 32824 | 42 | 48 | 35 | 195 | 178 | 121 |
| 35639 | 23 | 31 | 16 | 148 | 119 | 88 |
| 33669 | 1,328 | 1,325 | 851 | 602 | 557 | 429 |
| 51796 | 1,093 | 1,292 | 627 | 2,411 | 1,897 | 1,759 |
| 53312 | 420 | 359 | 375 | 1,569 | 1,462 | 840 |
| 37244 | 135 | 120 | 85 | 8,654 | 6,181 | 8,537 |
| 38577 | 378 | 390 | 251 | 1,175 | 982 | 1,156 |
| 38462 | 2,088 | 2,328 | 888 | 5,649 | 4,045 | 5,418 |
| 38740 | 269 | 284 | 171 | 1,111 | 879 | 958 |
| 37380 | 111 | 106 | 80 | 265 | 195 | 222 |
| 36707 | 564 | 445 | 599 | 5,874 | 5,908 | 2,726 |
| 39706 | 234 | 233 | 130 | 1,341 | 980 | 1,126 |
| 40873 | 1,134 | 1,106 | 699 | 212 | 179 | 135 |
| 39251 | 1,121 | 716 | 880 | 466 | 454 | 304 |
| 39325 | 349 | 344 | 280 | 8,639 | 7,278 | 6,545 |
| 39908 | 448 | 411 | 355 | 928 | 886 | 539 |
| 39663 | 232 | 183 | 177 | 149 | 142 | 83 |
| 43693 | 12,137 | 11,474 | 6,356 | 1,209 | 660 | 1,716 |
| 42104 | 211 | 218 | 105 | 1,455 | 1,062 | 1,324 |
| 44940 | 850 | 675 | 589 | 1,654 | 1,246 | 1,497 |
| 46703 | 598 | 654 | 459 | 1,208 | 1,084 | 775 |
| 46751 | 253 | 265 | 145 | 1,286 | 983 | 1,133 |
| 48204 | 443 | 417 | 308 | 1,639 | 1,249 | 1,441 |
| 47384 | 431 | 425 | 263 | 824 | 649 | 693 |
| 47879 | 263 | 239 | 196 | 877 | 798 | 678 |
| 47189 | 2,549 | 2,127 | 2,116 | 4,481 | 3,741 | 3,212 |
| 48876 | 251,438 | 237,070 | 185,469 | 144,998 | 149,888 | 62,407 |
| 49463 | 142,112 | 121,188 | 99,938 | 75,330 | 82,045 | 36,158 |
| 48444 | 1,533 | 1,725 | 854 | 563 | 539 | 528 |
| 48583 | 1,229 | 1,179 | 768 | 5,192 | 4,869 | 2,724 |
| 50856 | 1,266 | 1,073 | 1,022 | 248 | 174 | 207 |
| 104501 | 216 | 225 | 139 | 825 | 746 | 519 |
| 100503 | 1,129 | 1,221 | 721 | 2,388 | 2,054 | 2,403 |
| 104580 | 789 | 722 | 536 | 3,041 | 2,292 | 2,471 |
| 104818 | 7,009 | 6,483 | 4,557 | 11,660 | 9,649 | 9,972 |
| 101695 | 821 | 671 | 648 | 1,279 | 1,084 | 883 |
| 102089 | 1,090 | 1,049 | 797 | 2,016 | 1,635 | 1,548 |
| 102131 | 2,809 | 2,816 | 1,594 | 1,299 | 1,023 | 1,196 |
| 102411 | 2,853 | 2,896 | 1,843 | 1,896 | 1,489 | 1,525 |
| 102543 | 1,353 | 1,363 | 992 | 664 | 565 | 587 |
| 102695 | 523 | 658 | 285 | 3,131 | 2,205 | 2,699 |
| 102709 | 3,946 | 4,244 | 2,551 | 12,670 | 10,249 | 11,574 |
| 103155 | 8,688 | 8,649 | 6,161 | 4,937 | 3,753 | 4,122 |
| 103194 | 914 | 852 | 649 | 1,851 | 1,638 | 1,197 |
| 103640 | 523 | 658 | 285 | 3,131 | 2,205 | 2,699 |
| 103799 | 1,066 | 920 | 874 | 3,503 | 2,646 | 2,920 |
| 104198 | 72,770 | 69,975 | 58,326 | 34,504 | 29,513 | 27,598 |
| 109038 | 7,828 | 7,332 | 5,230 | 30,308 | 22,853 | 25,738 |
| 109085 | 502 | 526 | 339 | 2,968 | 2,344 | 2,822 |
| 105031 | 960 | 1,021 | 515 | 2,055 | 1,557 | 2,082 |
| 105038 | 357 | 320 | 293 | 1,259 | 965 | 1,133 |
| 109185 | 8,584 | 8,966 | 5,016 | 3,367 | 2,680 | 2,641 |
| 109204 | 928 | 1,080 | 517 | 11,214 | 8,017 | 10,250 |
| 109332 | 311 | 270 | 201 | 1,152 | 1,049 | 785 |
| 109461 | 1,687 | 1,776 | 893 | 4,341 | 2,699 | 4,686 |
| 105776 | 210 | 167 | 197 | 2,025 | 1,932 | 1,054 |
| 105976 | 3,102 | 3,155 | 1,846 | 7,183 | 5,721 | 5,768 |
| 106144 | 1,509 | 1,678 | 978 | 3,905 | 3,225 | 3,240 |
| 106186 | 549 | 579 | 338 | 1,074 | 882 | 943 |
| 106196 | 1,346 | 1,340 | 661 | 345 | 285 | 227 |
| 106275 | 4,315 | 2,261 | 4,949 | 8,641 | 6,541 | 8,746 |
| 106438 | 1,037 | 1,172 | 676 | 2,380 | 1,947 | 1,903 |
| 106447 | 220 | 164 | 236 | 1,061 | 1,136 | 468 |
| 106463 | 299 | 308 | 211 | 937 | 822 | 630 |
| 106743 | 1,136 | 1,098 | 713 | 2,717 | 1,951 | 2,522 |
| 107076 | 1,009 | 959 | 632 | 1,839 | 1,489 | 1,307 |
| 107309 | 1,465 | 1,335 | 932 | 678 | 527 | 499 |
| 107379 | 1,948 | 1,638 | 1,496 | 505 | 319 | 582 |
| 107401 | 145 | 113 | 101 | 2,026 | 1,776 | 1,287 |
| 107433 | 397 | 429 | 233 | 1,133 | 990 | 836 |
| 107492 | 1,258 | 1,281 | 840 | 2,770 | 2,323 | 2,392 |
| 107789 | 2,235 | 1,880 | 1,465 | 6,004 | 5,770 | 2,830 |
| 107810 | 978 | 800 | 804 | 283 | 224 | 241 |
| 107916 | 318 | 235 | 257 | 1,365 | 1,198 | 1,021 |
| 108033 | 2,997 | 3,190 | 1,609 | 1,670 | 1,258 | 1,620 |
| 108039 | 1,484 | 1,343 | 1,079 | 2,970 | 2,465 | 2,406 |
| 108192 | 9,235 | 8,501 | 5,694 | 17,374 | 13,330 | 16,003 |
| 108218 | 824 | 742 | 470 | 1,566 | 1,269 | 1,294 |
| 108424 | 314 | 309 | 181 | 1,114 | 877 | 952 |
| 108742 | 276 | 276 | 205 | 1,544 | 1,169 | 1,430 |
| 108748 | 1,931 | 1,820 | 1,070 | 618 | 466 | 515 |
| 108751 | 1,438 | 1,589 | 681 | 219 | 188 | 149 |
| 108828 | 1,719 | 1,654 | 1,165 | 5,139 | 3,945 | 4,743 |
| 108884 | 1,254 | 1,267 | 766 | 529 | 453 | 399 |
| 96747 | 524 | 571 | 327 | 2,974 | 2,202 | 2,723 |
| 96877 | 1,882 | 1,980 | 1,351 | 844 | 606 | 831 |
| 96955 | 2,831 | 2,789 | 2,054 | 4,588 | 4,352 | 2,864 |
| 100328 | 1,826 | 2,017 | 1,099 | 388 | 217 | 410 |
| 97853 | 500 | 430 | 394 | 979 | 725 | 1,115 |
| 98275 | 517 | 603 | 270 | 1,785 | 1,296 | 1,499 |
| 98392 | 1,951 | 1,648 | 1,176 | 279 | 205 | 314 |
| 98462 | 902 | 969 | 562 | 2,513 | 1,981 | 1,756 |
| 98481 | 877 | 921 | 514 | 1,578 | 1,296 | 1,365 |
| 98688 | 3,475 | 2,916 | 2,877 | 7,940 | 5,750 | 7,361 |
| 98857 | 2,078 | 1,802 | 1,572 | 4,815 | 3,704 | 4,603 |
| 98878 | 1,727 | 1,549 | 1,135 | 3,585 | 2,727 | 2,966 |
| 98980 | 446 | 413 | 350 | 1,448 | 1,097 | 1,312 |
| 99052 | 3,751 | 3,567 | 2,359 | 8,280 | 7,634 | 4,536 |
| 99084 | 1,611 | 1,515 | 1,088 | 3,623 | 2,903 | 3,222 |
| 99210 | 1,987 | 1,802 | 1,384 | 1,262 | 1,103 | 991 |
| 99346 | 289 | 338 | 179 | 1,176 | 832 | 1,011 |
| 99673 | 523 | 489 | 357 | 1,077 | 869 | 1,044 |
| 99684 | 140 | 130 | 92 | 216 | 204 | 186 |
| 99765 | 865 | 803 | 596 | 1,822 | 1,393 | 1,510 |
| 99849 | 910 | 760 | 570 | 2,151 | 1,825 | 1,516 |
| 99963 | 952 | 971 | 642 | 2,101 | 1,629 | 1,665 |
| 85177 | 1,358 | 1,407 | 819 | 423 | 346 | 331 |
| 85213 | 514 | 510 | 247 | 1,106 | 873 | 966 |
| 85351 | 263 | 154 | 262 | 821 | 807 | 316 |
| 76327 | 823 | 765 | 504 | 1,678 | 1,237 | 1,313 |
| 76524 | 1,041 | 993 | 685 | 3,189 | 2,415 | 2,937 |
| 76549 | 2,928 | 2,661 | 2,159 | 4,672 | 3,416 | 4,313 |
| 77436 | 612 | 715 | 354 | 2,548 | 1,881 | 2,137 |
| 77536 | 112 | 128 | 69 | 2,301 | 1,441 | 2,601 |
| 85578 | 2,807 | 4,246 | 564 | 6 | 4 | 10 |
| 85656 | 2,200 | 1,824 | 1,369 | 4,253 | 3,351 | 3,624 |
| 86125 | 34 | 39 | 39 | 238 | 176 | 180 |
| 78195 | 1,918 | 1,914 | 1,707 | 669 | 591 | 608 |
| 78289 | 2,054 | 1,801 | 1,533 | 5,382 | 5,146 | 2,808 |
| 78310 | 471 | 447 | 273 | 1,620 | 1,206 | 1,377 |
| 78350 | 197 | 226 | 101 | 1,193 | 866 | 1,088 |
| 78794 | 467 | 421 | 254 | 658 | 522 | 540 |
| 79046 | 443 | 462 | 231 | 1,045 | 828 | 1,003 |
| 79455 | 295 | 274 | 201 | 1,407 | 1,133 | 1,031 |
| 79478 | 5,563 | 5,583 | 4,269 | 3,307 | 2,733 | 3,051 |
| 79659 | 479 | 464 | 367 | 941 | 857 | 614 |
| 79672 | 4,008 | 4,291 | 2,816 | 517 | 416 | 441 |
| 79817 | 1,923 | 1,836 | 1,310 | 939 | 799 | 767 |
| 79995 | 2,211 | 2,312 | 1,395 | 1,069 | 804 | 837 |
| 80154 | 1,593 | 1,502 | 950 | 2,985 | 2,333 | 2,625 |
| 80190 | 871 | 878 | 559 | 1,701 | 1,395 | 1,425 |
| 80221 | 1,914 | 1,757 | 1,219 | 4,925 | 3,734 | 3,886 |
| 80584 | 2,203 | 3,047 | 307 | 3 | 4 | 3 |
| 80617 | 585 | 597 | 322 | 1,385 | 1,169 | 1,163 |
| 81350 | 1,594 | 1,520 | 1,086 | 590 | 516 | 415 |
| 81875 | 2,302 | 2,004 | 1,688 | 9,763 | 7,717 | 8,385 |
| 81901 | 1,122 | 1,095 | 779 | 290 | 185 | 257 |
| 82026 | 1,155 | 1,075 | 739 | 409 | 372 | 281 |
| 82840 | 565 | 515 | 437 | 113 | 98 | 86 |
| 83839 | 6,670 | 6,222 | 4,235 | 19,429 | 15,443 | 15,740 |
| 84036 | 1,372 | 996 | 1,194 | 3,425 | 2,735 | 2,886 |
| 84049 | 1,340 | 1,237 | 784 | 783 | 764 | 517 |
| 84078 | 1,282 | 1,052 | 1,053 | 10,079 | 8,162 | 7,338 |
| 84745 | 1,701 | 1,692 | 1,012 | 7,516 | 5,507 | 7,473 |
| 84910 | 418 | 394 | 278 | 1,946 | 1,543 | 1,777 |
| 95460 | 552 | 377 | 414 | 1,046 | 974 | 787 |
| 95466 | 1,177 | 1,099 | 704 | 713 | 538 | 576 |
| 86394 | 373 | 299 | 275 | 1,119 | 967 | 956 |
| 86486 | 1,368 | 1,320 | 860 | 3,639 | 2,938 | 3,019 |
| 95656 | 5,769 | 5,454 | 3,090 | 1,576 | 1,264 | 1,222 |
| 86840 | 2,348 | 2,245 | 1,723 | 5,052 | 4,032 | 5,077 |
| 86993 | 1,335 | 1,152 | 994 | 537 | 411 | 478 |
| 95850 | 749 | 753 | 466 | 1,773 | 1,492 | 1,349 |
| 87833 | 811 | 692 | 544 | 1,708 | 1,302 | 1,541 |
| 87973 | 716 | 662 | 539 | 2,073 | 1,646 | 1,665 |
| 96027 | 1,045 | 896 | 821 | 2,447 | 1,998 | 1,770 |
| 96048 | 520 | 683 | 250 | 5,154 | 3,996 | 5,198 |
| 96217 | 1,347 | 1,204 | 866 | 3,831 | 3,284 | 2,383 |
| 88222 | 1,114 | 801 | 1,185 | 16,735 | 16,684 | 8,196 |
| 88728 | 514 | 532 | 288 | 2,244 | 1,653 | 1,877 |
| 88905 | 2,516 | 2,319 | 1,816 | 4,360 | 3,653 | 3,775 |
| 89178 | 6,390 | 6,586 | 3,499 | 866 | 569 | 1,203 |
| 89402 | 1,609 | 1,349 | 1,267 | 4,013 | 2,742 | 3,832 |
| 89436 | 2,575 | 2,522 | 1,523 | 6,312 | 4,980 | 5,590 |
| 89535 | 834 | 656 | 697 | 287 | 232 | 245 |
| 90296 | 2,628 | 2,503 | 1,811 | 7,042 | 5,277 | 5,741 |
| 90525 | 3,031 | 2,831 | 1,825 | 8,834 | 7,050 | 6,507 |
| 90614 | 539 | 520 | 293 | 1,563 | 1,162 | 1,535 |
| 90806 | 10,507 | 10,427 | 6,905 | 6,592 | 5,514 | 5,440 |
| 92416 | 139 | 194 | 53 | 250 | 253 | 152 |
| 92430 | 2,742 | 2,460 | 2,095 | 1,614 | 1,332 | 1,269 |
| 94197 | 1,145 | 965 | 975 | 7,938 | 6,207 | 6,136 |
| 94269 | 224 | 176 | 182 | 1,731 | 1,642 | 868 |
| 94316 | 141 | 131 | 135 | 832 | 924 | 514 |
| 94760 | 3,117 | 2,862 | 2,201 | 10,194 | 7,640 | 8,667 |
| 94765 | 724 | 729 | 464 | 1,437 | 1,061 | 1,287 |
| 94862 | 2,058 | 1,799 | 1,167 | 9,721 | 7,056 | 9,156 |
| 58051 | 22,284 | 25,296 | 14,224 | 58,622 | 51,092 | 42,007 |
| 54208 | 953 | 697 | 1,016 | 10,021 | 7,182 | 11,125 |
| 58346 | 175 | 212 | 110 | 1,978 | 1,431 | 1,987 |
| 58417 | 1,983 | 2,011 | 1,371 | 4,661 | 3,986 | 3,510 |
| 58513 | 25,521 | 29,349 | 16,341 | 68,140 | 59,181 | 48,028 |
| 54421 | 21,323 | 24,391 | 13,731 | 56,348 | 49,115 | 39,967 |
| 54564 | 502 | 525 | 350 | 916 | 825 | 665 |
| 54987 | 16,179 | 18,390 | 10,399 | 42,932 | 37,374 | 30,755 |
| 55599 | 2,298 | 2,434 | 1,562 | 4,835 | 3,966 | 4,158 |
| 56103 | 17,269 | 19,591 | 11,115 | 45,740 | 39,329 | 32,600 |
| 56490 | 9,188 | 11,734 | 5,259 | 28,774 | 24,417 | 17,712 |
| 56833 | 12,374 | 14,105 | 7,964 | 33,021 | 28,648 | 23,809 |
| 57319 | 297 | 263 | 286 | 4,872 | 4,960 | 1,963 |
| 75044 | 491 | 483 | 418 | 1,222 | 1,222 | 722 |
| 75085 | 1,156 | 1,151 | 768 | 2,117 | 1,753 | 1,541 |
| 66328 | 1,181 | 1,384 | 524 | 554 | 521 | 381 |
| 67680 | 300 | 279 | 209 | 136 | 126 | 130 |
| 75389 | 1,775 | 1,745 | 1,043 | 654 | 489 | 539 |
| 75605 | 558 | 485 | 400 | 836 | 676 | 754 |
| 75956 | 428 | 457 | 226 | 4,666 | 3,413 | 4,022 |
| 67771 | 327 | 216 | 308 | 11,416 | 10,948 | 5,196 |
| 67772 | 406 | 278 | 399 | 10,997 | 10,908 | 5,036 |
| 67777 | 1,249 | 468 | 1,632 | 50 | 63 | 39 |
| 67838 | 814 | 858 | 398 | 6,937 | 5,029 | 5,668 |
| 67931 | 619 | 467 | 482 | 1,291 | 1,246 | 659 |
| 68119 | 87 | 91 | 56 | 161 | 161 | 100 |
| 68578 | 613 | 547 | 410 | 251 | 204 | 176 |
| 69377 | 71 | 68 | 84 | 1,313 | 1,122 | 1,025 |
| 69531 | 4,812 | 4,199 | 3,441 | 1,671 | 1,280 | 1,384 |
| 69825 | 527 | 487 | 391 | 237 | 193 | 215 |
| 70047 | 372 | 285 | 297 | 643 | 536 | 428 |
| 70070 | 2,323 | 2,196 | 1,278 | 7,142 | 5,844 | 5,113 |
| 71798 | 1,304 | 1,204 | 816 | 2,138 | 1,820 | 1,531 |
| 72186 | 420 | 295 | 390 | 3,454 | 3,253 | 2,156 |
| 72880 | 280 | 216 | 196 | 1,139 | 1,074 | 544 |
| 73649 | 285 | 237 | 190 | 807 | 641 | 675 |
| 74249 | 173 | 146 | 131 | 121 | 107 | 68 |
| 58880 | 2,999 | 2,216 | 2,764 | 1,766 | 1,418 | 1,664 |
| 58930 | 847 | 752 | 554 | 1,745 | 1,385 | 1,389 |
| 65363 | 1,160 | 1,018 | 766 | 2,295 | 1,678 | 2,065 |
| 65847 | 2,139 | 1,564 | 1,735 | 4,884 | 4,231 | 3,186 |
| 61062 | 438 | 493 | 287 | 1,275 | 1,181 | 643 |
| 61076 | 265 | 221 | 160 | 1,814 | 1,462 | 1,508 |
| 61958 | 777 | 707 | 528 | 1,675 | 1,453 | 998 |
| 62434 | 563 | 426 | 460 | 301 | 286 | 291 |
| 62438 | 302 | 346 | 191 | 1,718 | 1,484 | 1,591 |
| 62602 | 76 | 60 | 53 | 402 | 339 | 227 |
| 62832 | 982 | 1,001 | 650 | 2,560 | 2,180 | 1,741 |
| 62927 | 376 | 432 | 202 | 1,240 | 1,077 | 1,053 |
| 63094 | 1,382 | 1,418 | 952 | 691 | 530 | 598 |
| 63271 | 146 | 197 | 77 | 1,362 | 1,110 | 1,124 |
| 63323 | 786 | 847 | 447 | 1,970 | 1,515 | 1,705 |
| 63707 | 166 | 133 | 126 | 1,683 | 868 | 2,187 |
| 64023 | 491 | 471 | 342 | 1,332 | 952 | 1,458 |
| 64466 | 685 | 559 | 443 | 1,576 | 1,330 | 1,216 |
| 64559 | 1,111 | 1,015 | 738 | 1,660 | 1,417 | 1,243 |
| 64984 | 153 | 160 | 84 | 2,284 | 2,135 | 890 |
| 65007 | 799 | 620 | 563 | 3,003 | 2,564 | 2,088 |
| 20616 | 426 | 367 | 232 | 1,468 | 1,186 | 1,054 |
| 13424 | 8 | 6 | 6 | 80 | 78 | 61 |
| 11941 | 36 | 27 | 37 | 1,292 | 1,187 | 823 |
| 15079 | 500 | 489 | 382 | 1,052 | 855 | 888 |
| 12734 | 565 | 464 | 358 | 1,345 | 1,141 | 1,011 |
| 31234 | 188 | 197 | 115 | 1,076 | 815 | 1,255 |
| 32614 | 1,127 | 1,103 | 762 | 694 | 677 | 290 |
| 15980 | 12 | 15 | 9 | 2,432 | 2,523 | 980 |
| 246811 | 165 | 163 | 122 | 636 | 611 | 377 |
| 248975 | 9,826 | 8,339 | 8,110 | 2,620 | 2,168 | 2,570 |
| 245011 | 145 | 127 | 85 | 389 | 352 | 264 |
| 247336 | 561 | 495 | 397 | 1,398 | 1,063 | 1,275 |
| 247692 | 1,395 | 1,187 | 932 | 709 | 589 | 507 |
| 246900 | 429 | 349 | 300 | 1,049 | 858 | 890 |
| 246669 | 51 | 43 | 46 | 243 | 183 | 205 |
| 245974 | 306 | 157 | 375 | 1,311 | 1,258 | 778 |
| 247366 | 154 | 142 | 113 | 411 | 293 | 343 |
| 247767 | 282 | 260 | 182 | 16 | 16 | 7 |
| 245931 | 309 | 372 | 186 | 1,522 | 1,027 | 1,527 |
| 248021 | 1,194 | 1,064 | 825 | 2,026 | 1,582 | 1,429 |
| 245744 | 822 | 736 | 531 | 1,827 | 1,537 | 1,321 |
| 246417 | 251 | 242 | 152 | 949 | 752 | 797 |
| 248043 | 36 | 31 | 20 | 2,125 | 2,082 | 935 |
| 248075 | 370 | 330 | 229 | 1,581 | 1,288 | 1,400 |
| 248429 | 375 | 435 | 202 | 154 | 129 | 131 |
| 248498 | 16 | 4 | 7 | 113 | 105 | 99 |
| 245082 | 443 | 386 | 242 | 1,794 | 1,125 | 2,331 |
| 245902 | 11 | 13 | 7 | 144 | 118 | 108 |
| 269678 | 248 | 213 | 194 | 1,716 | 1,902 | 733 |
| 269446 | 1,666 | 1,473 | 979 | 3,880 | 3,108 | 3,074 |
| 249522 | 1,023 | 1,033 | 560 | 8,517 | 6,707 | 6,851 |
| 250312 | 724 | 717 | 478 | 2,404 | 1,679 | 2,328 |
| 250943 | 587 | 586 | 354 | 1,266 | 1,006 | 982 |
| 252003 | 4,971 | 3,386 | 4,821 | 8,725 | 8,329 | 4,592 |
| 251667 | 148 | 84 | 180 | 5,320 | 5,192 | 2,831 |
| 253099 | 396 | 380 | 254 | 1,077 | 1,043 | 615 |
| 252031 | 1,140 | 1,054 | 808 | 326 | 182 | 254 |
| 253127 | 1,716 | 1,149 | 1,645 | 371 | 265 | 381 |
| 254425 | 896 | 1,016 | 521 | 1,935 | 1,492 | 1,535 |
| 252226 | 267 | 310 | 154 | 824 | 627 | 684 |
| 254916 | 97 | 89 | 90 | 1,086 | 912 | 784 |
| 253334 | 751 | 594 | 614 | 2,320 | 2,290 | 1,270 |
| 252188 | 372 | 381 | 274 | 2,315 | 1,977 | 1,484 |
| 254393 | 574 | 477 | 360 | 1,161 | 878 | 1,143 |
| 252515 | 1,065 | 985 | 757 | 496 | 424 | 325 |
| 254125 | 266 | 290 | 172 | 1,208 | 856 | 1,004 |
| 254745 | 1,528 | 1,518 | 892 | 4,873 | 3,385 | 4,676 |
| 253279 | 23 | 20 | 16 | 2,051 | 1,944 | 887 |
| 256096 | 4,998 | 3,231 | 4,933 | 88,081 | 78,754 | 47,741 |
| 255785 | 282 | 277 | 201 | 127 | 104 | 98 |
| 256797 | 244 | 264 | 139 | 1,336 | 1,012 | 1,193 |
| 257204 | 2,069 | 1,769 | 1,659 | 11,020 | 9,714 | 6,645 |
| 257164 | 1,949 | 1,905 | 1,220 | 5,641 | 4,613 | 4,060 |
| 256337 | 256 | 301 | 158 | 1,166 | 902 | 967 |
| 256253 | 205 | 198 | 129 | 94 | 80 | 63 |
| 257710 | 271 | 266 | 196 | 591 | 450 | 461 |
| 255304 | 18 | 21 | 23 | 1,562 | 1,425 | 915 |
| 255780 | 581 | 568 | 311 | 2,340 | 1,703 | 2,052 |
| 258992 | 298 | 339 | 167 | 1,554 | 1,229 | 1,347 |
| 258342 | 760 | 526 | 604 | 46 | 15 | 94 |
| 260707 | 315 | 290 | 170 | 1,086 | 868 | 1,078 |
| 258472 | 316 | 255 | 252 | 2,159 | 2,098 | 1,084 |
| 259317 | 2,642 | 2,003 | 2,452 | 1,362 | 941 | 1,366 |
| 259267 | 1,436 | 1,439 | 811 | 623 | 528 | 466 |
| 260943 | 342 | 332 | 193 | 891 | 698 | 680 |
| 261299 | 1,154 | 726 | 1,068 | 11,181 | 11,100 | 6,875 |
| 263010 | 586 | 509 | 470 | 2,606 | 1,734 | 2,914 |
| 262372 | 16,503 | 16,334 | 10,351 | 6,645 | 7,349 | 2,571 |
| 262519 | 16,503 | 16,334 | 10,351 | 6,645 | 7,349 | 2,571 |
| 260972 | 66 | 47 | 72 | 1,495 | 968 | 1,802 |
| 261302 | 251,342 | 239,213 | 187,956 | 146,671 | 153,730 | 62,914 |
| 261750 | 251,342 | 239,213 | 187,956 | 146,671 | 153,730 | 62,914 |
| 262841 | 17,105 | 18,031 | 12,511 | 9,354 | 9,145 | 3,502 |
| 262903 | 17,105 | 18,031 | 12,511 | 9,354 | 9,145 | 3,502 |
| 261677 | 70,290 | 72,564 | 49,899 | 34,963 | 38,154 | 14,390 |
| 262161 | 70,290 | 72,564 | 49,899 | 34,963 | 38,154 | 14,390 |
| 262010 | 158,952 | 166,025 | 127,829 | 95,647 | 111,467 | 40,842 |
| 261189 | 158,952 | 166,025 | 127,829 | 95,647 | 111,467 | 40,842 |
| 262935 | 158,952 | 166,025 | 127,829 | 95,647 | 111,467 | 40,842 |
| 262068 | 176 | 183 | 121 | 35 | 32 | 29 |
| 262227 | 195,663 | 187,587 | 146,064 | 107,904 | 118,134 | 45,704 |
| 261455 | 195,663 | 187,587 | 146,064 | 107,904 | 118,134 | 45,704 |
| 262211 | 1,148 | 996 | 704 | 2,827 | 2,036 | 2,751 |
| 264161 | 650 | 586 | 426 | 1,367 | 1,364 | 942 |
| 264935 | 306 | 306 | 171 | 1,623 | 1,222 | 1,442 |
| 264793 | 293 | 292 | 124 | 1,423 | 1,053 | 1,144 |
| 264798 | 359 | 395 | 270 | 1,418 | 1,266 | 549 |
| 263163 | 385 | 340 | 247 | 951 | 721 | 850 |
| 264732 | 238 | 254 | 201 | 938 | 835 | 546 |
| 265006 | 433 | 381 | 249 | 3,475 | 2,861 | 2,773 |
| 265386 | 1,223 | 1,454 | 469 | 1,886 | 1,406 | 1,708 |
| 266098 | 285 | 277 | 155 | 1,482 | 1,363 | 794 |
| 266499 | 289 | 328 | 175 | 1,082 | 675 | 1,153 |
| 266480 | 665 | 640 | 380 | 1,091 | 997 | 606 |
| 268102 | 480 | 531 | 244 | 1,104 | 943 | 876 |
| 268459 | 1,426 | 1,450 | 849 | 2,387 | 2,050 | 1,838 |
| 268380 | 1,902 | 1,782 | 1,245 | 5,975 | 5,140 | 5,300 |
| 268530 | 433 | 371 | 316 | 792 | 685 | 700 |
| 268597 | 380 | 334 | 227 | 1,217 | 990 | 1,023 |
| 268342 | 119 | 76 | 105 | 1,467 | 1,512 | 594 |
| 358514 | 266 | 287 | 135 | 1,575 | 1,153 | 1,391 |
| 358516 | 1,322 | 1,606 | 728 | 23 | 13 | 26 |
| 358554 | 3,261 | 3,655 | 1,983 | 7,260 | 5,804 | 5,911 |
| 358559 | 597 | 486 | 483 | 1,904 | 1,879 | 1,137 |
| 358563 | 716 | 704 | 422 | 1,475 | 1,243 | 1,363 |
| 358569 | 2,023 | 2,271 | 1,139 | 396 | 332 | 303 |
| 354012 | 142 | 145 | 96 | 2,742 | 2,218 | 2,819 |
| 358589 | 489 | 507 | 291 | 297 | 293 | 177 |
| 358614 | 1,996 | 2,220 | 1,294 | 4,163 | 3,234 | 3,742 |
| 358621 | 378 | 346 | 295 | 228 | 180 | 136 |
| 358629 | 300 | 310 | 149 | 3,188 | 2,769 | 2,599 |
| 358636 | 884 | 1,037 | 496 | 10,859 | 7,749 | 10,037 |
| 354048 | 306 | 281 | 235 | 119 | 102 | 110 |
| 354075 | 1,187 | 850 | 1,138 | 439 | 343 | 499 |
| 354084 | 378 | 377 | 207 | 1,208 | 951 | 1,015 |
| 354105 | 1,730 | 1,663 | 1,144 | 524 | 324 | 571 |
| 354114 | 1,074 | 1,470 | 500 | 34 | 32 | 35 |
| 354122 | 366 | 333 | 242 | 204 | 181 | 106 |
| 354138 | 332 | 318 | 240 | 1,371 | 1,362 | 560 |
| 354181 | 580 | 576 | 391 | 1,346 | 1,027 | 1,170 |
| 354182 | 671 | 603 | 608 | 2,418 | 1,839 | 2,518 |
| 354183 | 1,131 | 966 | 799 | 3,139 | 2,668 | 2,631 |
| 354233 | 6,494 | 5,288 | 5,017 | 3,108 | 2,480 | 2,712 |
| 354295 | 1,322 | 1,298 | 1,037 | 8,358 | 5,288 | 9,595 |
| 354312 | 1,549 | 1,325 | 1,130 | 2,398 | 2,224 | 1,741 |
| 354319 | 558 | 545 | 403 | 1,498 | 1,179 | 1,083 |
| 354344 | 2,432 | 2,664 | 1,519 | 5,843 | 4,565 | 4,810 |
| 354434 | 1,686 | 1,732 | 1,031 | 3,562 | 2,776 | 2,943 |
| 354460 | 2,644 | 2,615 | 1,717 | 261 | 227 | 214 |
| 354471 | 2,392 | 3,042 | 855 | 1,284 | 1,047 | 1,036 |
| 354483 | 612 | 456 | 604 | 2,043 | 1,633 | 1,843 |
| 354528 | 11,928 | 10,995 | 8,438 | 24,361 | 21,451 | 19,733 |
| 354540 | 1,490 | 1,578 | 827 | 3,241 | 2,343 | 3,032 |
| 354571 | 1,921 | 1,944 | 1,293 | 327 | 247 | 309 |
| 354579 | 2,420 | 2,136 | 1,953 | 4,357 | 3,704 | 3,820 |
| 354666 | 595 | 652 | 390 | 1,298 | 1,002 | 1,120 |
| 354674 | 2,268 | 2,023 | 1,715 | 1,256 | 996 | 1,077 |
| 354736 | 309 | 286 | 256 | 4,199 | 3,733 | 2,543 |
| 354807 | 272 | 279 | 215 | 767 | 682 | 460 |
| 354844 | 3,321 | 3,967 | 1,361 | 1,393 | 991 | 1,329 |
| 358649 | 1,142 | 1,046 | 895 | 4,151 | 3,804 | 2,907 |
| 358650 | 379 | 409 | 242 | 3,994 | 2,769 | 4,612 |
| 358665 | 358 | 364 | 227 | 1,218 | 1,048 | 854 |
| 358682 | 465 | 432 | 272 | 1,890 | 1,468 | 1,556 |
| 358688 | 131 | 142 | 91 | 2,310 | 1,919 | 1,893 |
| 358700 | 5,192 | 4,823 | 3,072 | 699 | 478 | 770 |
| 354872 | 559 | 604 | 418 | 1,838 | 1,285 | 2,111 |
| 354877 | 266 | 287 | 135 | 1,575 | 1,153 | 1,391 |
| 354890 | 13,262 | 13,494 | 7,065 | 5,803 | 4,512 | 4,955 |
| 354909 | 1,277 | 1,321 | 968 | 2,504 | 1,919 | 1,862 |
| 354926 | 512 | 538 | 335 | 1,805 | 1,578 | 1,731 |
| 354935 | 475 | 425 | 366 | 1,216 | 1,382 | 583 |
| 354939 | 794 | 866 | 499 | 3,011 | 2,377 | 2,692 |
| 354980 | 1,193 | 1,150 | 803 | 229 | 166 | 213 |
| 354996 | 538 | 599 | 387 | 6,020 | 4,434 | 5,486 |
| 355019 | 1,078 | 1,095 | 747 | 274 | 192 | 255 |
| 355044 | 527 | 478 | 298 | 1,095 | 929 | 782 |
| 355061 | 3,491 | 3,723 | 1,973 | 1,612 | 1,215 | 1,565 |
| 355096 | 912 | 847 | 506 | 1,717 | 1,344 | 1,575 |
| 355134 | 2,480 | 3,082 | 1,752 | 1,782 | 1,485 | 1,298 |
| 355167 | 235 | 277 | 106 | 418 | 405 | 254 |
| 355226 | 5,560 | 4,845 | 3,981 | 15,918 | 14,826 | 9,728 |
| 355228 | 1,157 | 1,028 | 874 | 332 | 230 | 253 |
| 355236 | 693 | 816 | 408 | 2,019 | 1,629 | 1,791 |
| 355260 | 4,277 | 4,016 | 2,953 | 7,253 | 5,740 | 5,813 |
| 355270 | 2,503 | 1,892 | 2,339 | 22,562 | 21,493 | 11,892 |
| 355276 | 2,497 | 1,846 | 2,518 | 8,775 | 9,220 | 3,392 |
| 355279 | 3,270 | 2,723 | 2,882 | 7,083 | 6,929 | 2,460 |
| 355284 | 3,779 | 2,884 | 3,890 | 7,852 | 8,351 | 3,280 |
| 355290 | 13,264 | 11,785 | 11,096 | 44,575 | 43,613 | 26,395 |
| 355303 | 2,127 | 1,691 | 2,074 | 4,429 | 4,790 | 1,891 |
| 355316 | 4,182 | 4,138 | 3,080 | 6,889 | 5,644 | 5,539 |
| 355317 | 1,855 | 1,781 | 1,415 | 4,654 | 4,067 | 3,573 |
| 355322 | 1,217 | 1,139 | 880 | 2,635 | 2,399 | 1,647 |
| 355358 | 1,215 | 1,431 | 825 | 367 | 318 | 306 |
| 355390 | 195 | 132 | 235 | 1,986 | 1,374 | 2,427 |
| 355394 | 5,021 | 5,373 | 3,235 | 1,297 | 1,024 | 1,189 |
| 355402 | 220 | 190 | 195 | 502 | 464 | 361 |
| 355404 | 324 | 274 | 262 | 1,546 | 1,210 | 1,214 |
| 355422 | 224 | 222 | 204 | 1,418 | 1,063 | 1,348 |
| 355436 | 538 | 677 | 250 | 1,060 | 720 | 1,094 |
| 355461 | 582 | 703 | 362 | 131 | 114 | 102 |
| 355471 | 1,226 | 1,285 | 854 | 227 | 181 | 174 |
| 355478 | 1,552 | 1,282 | 1,195 | 20,399 | 13,459 | 23,073 |
| 355526 | 10,407 | 8,944 | 7,629 | 1,849 | 1,220 | 1,804 |
| 355540 | 1,750 | 1,817 | 1,430 | 855 | 758 | 784 |
| 355627 | 835 | 730 | 617 | 1,628 | 1,392 | 1,370 |
| 355657 | 3,948 | 3,803 | 2,745 | 1,851 | 1,535 | 1,577 |
| 355691 | 933 | 1,298 | 711 | 663 | 584 | 495 |
| 355718 | 499 | 547 | 323 | 1,104 | 765 | 1,032 |
| 355740 | 876 | 880 | 509 | 2,604 | 1,935 | 2,292 |
| 355765 | 575 | 567 | 349 | 372 | 314 | 180 |
| 355777 | 645 | 751 | 426 | 2,404 | 2,194 | 1,307 |
| 355780 | 493 | 610 | 361 | 3,328 | 3,348 | 1,863 |
| 355825 | 2,497 | 2,614 | 1,483 | 7,309 | 5,739 | 6,242 |
| 355838 | 1,583 | 1,207 | 1,095 | 636 | 574 | 453 |
| 355847 | 1,219 | 1,277 | 1,118 | 4,523 | 3,722 | 2,750 |
| 355854 | 308 | 238 | 269 | 1,143 | 1,094 | 771 |
| 355864 | 1,115 | 920 | 1,080 | 15,428 | 14,555 | 7,172 |
| 355867 | 3,891 | 3,002 | 3,628 | 24,236 | 23,349 | 13,873 |
| 355871 | 893 | 920 | 562 | 7,207 | 5,868 | 6,097 |
| 355874 | 506 | 469 | 331 | 1,068 | 955 | 929 |
| 355885 | 428 | 403 | 283 | 1,726 | 1,436 | 1,416 |
| 355904 | 266 | 287 | 135 | 1,575 | 1,153 | 1,391 |
| 355921 | 8,746 | 8,060 | 6,163 | 19,203 | 17,871 | 10,831 |
| 355959 | 1,737 | 1,702 | 1,160 | 10,755 | 8,895 | 8,698 |
| 355964 | 163 | 178 | 167 | 125 | 134 | 77 |
| 355969 | 320 | 218 | 328 | 1,082 | 1,102 | 687 |
| 356015 | 2,217 | 2,141 | 1,391 | 15,197 | 9,740 | 17,473 |
| 356022 | 3,502 | 3,109 | 2,383 | 1,031 | 703 | 1,127 |
| 356024 | 1,542 | 1,768 | 1,117 | 4,054 | 3,562 | 2,654 |
| 356034 | 1,288 | 949 | 1,086 | 1,884 | 1,549 | 2,006 |
| 356048 | 545 | 449 | 470 | 1,434 | 1,224 | 1,194 |
| 356051 | 873 | 934 | 532 | 2,092 | 1,692 | 2,028 |
| 356052 | 333 | 521 | 81 | 3,168 | 3,075 | 1,845 |
| 356066 | 14,260 | 14,457 | 8,192 | 40,337 | 29,226 | 36,716 |
| 356101 | 506 | 390 | 427 | 2,238 | 2,160 | 1,390 |
| 356109 | 3,122 | 3,462 | 1,920 | 6,293 | 5,037 | 5,753 |
| 356120 | 884 | 1,037 | 496 | 10,859 | 7,749 | 10,037 |
| 356157 | 446 | 518 | 340 | 151 | 133 | 95 |
| 356207 | 1,212 | 1,180 | 681 | 2,544 | 1,931 | 2,457 |
| 356222 | 189 | 133 | 139 | 657 | 453 | 557 |
| 356236 | 847 | 769 | 693 | 343 | 287 | 206 |
| 356265 | 1,607 | 1,546 | 1,254 | 688 | 544 | 489 |
| 356330 | 1,470 | 1,290 | 1,091 | 2,268 | 1,983 | 1,523 |
| 356334 | 141 | 144 | 108 | 293 | 210 | 280 |
| 356341 | 1,450 | 1,120 | 1,196 | 2,538 | 2,497 | 1,431 |
| 356385 | 281 | 291 | 190 | 1,663 | 1,269 | 1,588 |
| 356387 | 261 | 216 | 197 | 1,336 | 960 | 1,461 |
| 356390 | 11,244 | 11,265 | 7,170 | 26,696 | 21,171 | 22,059 |
| 356403 | 294 | 299 | 222 | 2,043 | 1,682 | 1,606 |
| 356434 | 2,221 | 2,439 | 1,354 | 239 | 202 | 163 |
| 356462 | 1,920 | 2,036 | 1,200 | 592 | 453 | 573 |
| 356509 | 1,195 | 1,272 | 666 | 154 | 140 | 105 |
| 356517 | 487 | 482 | 336 | 1,786 | 1,628 | 1,301 |
| 356543 | 1,134 | 1,182 | 714 | 445 | 427 | 301 |
| 356565 | 946 | 995 | 621 | 1,664 | 1,706 | 836 |
| 356645 | 1,292 | 1,517 | 803 | 2,968 | 2,329 | 2,482 |
| 356682 | 1,434 | 1,430 | 807 | 4,076 | 3,384 | 2,718 |
| 356698 | 1,857 | 1,718 | 1,258 | 1,044 | 714 | 997 |
| 356729 | 1,632 | 1,850 | 932 | 3,627 | 2,691 | 3,334 |
| 356746 | 449 | 463 | 255 | 1,819 | 1,301 | 1,601 |
| 356776 | 426 | 501 | 254 | 8,200 | 6,798 | 6,825 |
| 356821 | 2,517 | 2,805 | 1,573 | 5,358 | 3,940 | 4,720 |
| 356838 | 4,634 | 4,710 | 3,039 | 1,781 | 1,579 | 1,382 |
| 356898 | 1,013 | 708 | 978 | 363 | 354 | 215 |
| 356936 | 284 | 232 | 218 | 1,067 | 1,086 | 634 |
| 356957 | 2,367 | 2,898 | 1,234 | 4 | 1 | 4 |
| 356961 | 1,718 | 2,337 | 953 | 3 | 4 | 1 |
| 356989 | 1,767 | 1,648 | 1,363 | 3,266 | 2,316 | 3,370 |
| 356997 | 628 | 503 | 538 | 9,590 | 9,530 | 3,735 |
| 357020 | 8,903 | 8,611 | 7,544 | 1,575 | 1,465 | 1,595 |
| 357062 | 1,651 | 1,417 | 1,757 | 10,751 | 10,859 | 3,748 |
| 357066 | 1,271 | 1,090 | 1,058 | 13,032 | 8,647 | 14,501 |
| 357075 | 1,798 | 1,412 | 1,920 | 13,949 | 12,454 | 9,742 |
| 357083 | 60 | 50 | 45 | 33 | 29 | 21 |
| 357090 | 5,901 | 4,345 | 6,408 | 28,017 | 30,681 | 10,831 |
| 357113 | 1,489 | 1,973 | 498 | 4 | 1 | 5 |
| 357114 | 1,659 | 2,265 | 843 | 214 | 160 | 136 |
| 357120 | 2,980 | 3,568 | 1,812 | 1,301 | 1,088 | 1,165 |
| 357133 | 328 | 304 | 222 | 1,613 | 1,298 | 1,399 |
| 357181 | 266 | 287 | 135 | 1,575 | 1,153 | 1,391 |
| 357240 | 1,473 | 1,655 | 642 | 12 | 16 | 17 |
| 357257 | 2,357 | 1,840 | 1,745 | 4,226 | 2,712 | 4,884 |
| 357263 | 264 | 219 | 181 | 3,208 | 2,722 | 2,088 |
| 357279 | 3,504 | 2,630 | 3,221 | 11,300 | 10,142 | 8,526 |
| 357287 | 796 | 671 | 507 | 1,464 | 1,168 | 1,265 |
| 357291 | 276 | 304 | 252 | 1,165 | 1,095 | 796 |
| 357320 | 498 | 506 | 411 | 1,007 | 875 | 555 |
| 357338 | 7,742 | 7,507 | 4,919 | 1,548 | 1,371 | 1,096 |
| 357360 | 1,482 | 1,510 | 892 | 392 | 357 | 227 |
| 357433 | 1,442 | 1,520 | 902 | 698 | 514 | 569 |
| 357450 | 1,199 | 1,277 | 778 | 3,065 | 2,330 | 2,621 |
| 357459 | 427 | 427 | 341 | 855 | 832 | 665 |
| 357473 | 930 | 896 | 596 | 1,881 | 1,410 | 1,601 |
| 357480 | 6,000 | 5,104 | 4,598 | 2,121 | 1,762 | 1,793 |
| 357530 | 933 | 850 | 707 | 3,212 | 2,365 | 3,401 |
| 357609 | 1,757 | 1,871 | 1,293 | 3,494 | 2,998 | 3,219 |
| 357610 | 2,442 | 2,546 | 1,896 | 1,058 | 844 | 1,056 |
| 357619 | 1,542 | 1,926 | 921 | 3,630 | 2,810 | 2,863 |
| 357639 | 823 | 768 | 562 | 2,006 | 1,473 | 1,757 |
| 357650 | 884 | 1,037 | 496 | 10,859 | 7,749 | 10,037 |
| 357653 | 1,371 | 1,267 | 1,123 | 820 | 645 | 579 |
| 357679 | 350 | 360 | 154 | 1,460 | 1,068 | 1,547 |
| 357680 | 784 | 807 | 489 | 2,430 | 1,980 | 2,418 |
| 357688 | 344 | 328 | 200 | 1,196 | 791 | 1,190 |
| 357698 | 3,519 | 2,981 | 2,905 | 14,183 | 14,609 | 6,910 |
| 357771 | 2,426 | 2,620 | 1,433 | 4,816 | 3,586 | 4,232 |
| 358068 | 122 | 179 | 13 | 3 | 4 | 6 |
| 358079 | 1,210 | 1,258 | 817 | 495 | 445 | 338 |
| 358085 | 2,917 | 3,083 | 1,824 | 627 | 469 | 618 |
| 358087 | 588 | 555 | 430 | 344 | 279 | 329 |
| 358121 | 571 | 489 | 474 | 1,618 | 1,812 | 825 |
| 358131 | 1,249 | 1,160 | 837 | 460 | 374 | 415 |
| 358133 | 4,244 | 4,770 | 2,534 | 421 | 340 | 328 |
| 358134 | 477 | 440 | 278 | 180 | 119 | 139 |
| 358138 | 963 | 877 | 653 | 26,139 | 26,540 | 10,777 |
| 358162 | 1,664 | 1,861 | 924 | 448 | 467 | 283 |
| 358188 | 2,165 | 2,217 | 1,287 | 4,533 | 3,508 | 3,546 |
| 358203 | 1,893 | 2,111 | 1,088 | 4,278 | 2,934 | 3,618 |
| 358228 | 2,773 | 2,466 | 2,059 | 6,687 | 4,514 | 7,555 |
| 358233 | 287 | 260 | 299 | 2,762 | 2,995 | 1,135 |
| 358238 | 184 | 179 | 193 | 5,697 | 3,975 | 8,722 |
| 358302 | 266 | 287 | 135 | 1,575 | 1,153 | 1,391 |
| 358313 | 200 | 176 | 156 | 133 | 92 | 85 |
| 358347 | 982 | 949 | 644 | 2,453 | 1,785 | 2,209 |
| 358371 | 454 | 356 | 428 | 918 | 787 | 894 |
| 358382 | 591 | 440 | 616 | 1,232 | 772 | 1,350 |
| 358399 | 1,907 | 1,781 | 1,393 | 107 | 111 | 88 |
| 358442 | 2,312 | 1,837 | 1,863 | 4,037 | 3,428 | 2,989 |
| 358454 | 42,928 | 41,711 | 27,342 | 24,386 | 19,471 | 19,778 |
| 358460 | 1,456 | 1,681 | 749 | 3,102 | 2,843 | 1,537 |
| 358511 | 884 | 1,037 | 496 | 10,859 | 7,749 | 10,037 |
| 358513 | 266 | 287 | 135 | 1,575 | 1,153 | 1,391 |
| 353782 | 2,217 | 2,435 | 1,083 | 3,586 | 3,522 | 2,302 |
| 353850 | 2,915 | 2,390 | 2,120 | 11,007 | 8,262 | 10,037 |
| 353885 | 341 | 312 | 232 | 1,104 | 946 | 861 |
| 348724 | 434 | 431 | 287 | 3,719 | 2,545 | 3,930 |
| 348798 | 9 | 14 | 5 | 4 | 3 | 1 |
| 348845 | 1,591 | 1,305 | 1,130 | 980 | 769 | 763 |
| 348971 | 1,385 | 1,307 | 830 | 3,510 | 2,746 | 2,949 |
| 349206 | 519 | 515 | 369 | 1,077 | 924 | 749 |
| 349296 | 2,212 | 2,901 | 781 | 152 | 109 | 106 |
| 349460 | 590 | 537 | 392 | 1,509 | 1,156 | 1,245 |
| 349531 | 321 | 341 | 280 | 164 | 150 | 98 |
| 349534 | 294 | 241 | 219 | 1,309 | 1,193 | 956 |
| 349723 | 1,438 | 1,529 | 975 | 4,604 | 3,466 | 5,577 |
| 349823 | 461 | 453 | 314 | 1,422 | 1,119 | 1,284 |
| 349858 | 217 | 226 | 84 | 952 | 755 | 747 |
| 349984 | 417 | 418 | 249 | 2,119 | 1,386 | 2,122 |
| 350114 | 505 | 430 | 416 | 4,097 | 3,700 | 2,648 |
| 350192 | 1,320 | 846 | 1,279 | 9,553 | 9,116 | 4,704 |
| 350207 | 783 | 665 | 690 | 291 | 173 | 446 |
| 350452 | 354 | 339 | 339 | 2,320 | 2,409 | 1,019 |
| 350499 | 1,124 | 964 | 968 | 20,094 | 19,816 | 7,706 |
| 350904 | 446 | 408 | 296 | 266 | 272 | 217 |
| 350924 | 469 | 379 | 407 | 1,718 | 1,521 | 1,137 |
| 350927 | 335 | 296 | 358 | 2,499 | 2,349 | 1,391 |
| 350943 | 555 | 542 | 369 | 1,565 | 1,185 | 1,342 |
| 351266 | 1,122 | 1,019 | 668 | 2,234 | 1,782 | 1,903 |
| 351305 | 815 | 796 | 521 | 2,801 | 2,004 | 2,557 |
| 351400 | 756 | 751 | 412 | 2,615 | 2,016 | 2,173 |
| 351487 | 136 | 114 | 114 | 731 | 673 | 396 |
| 351545 | 2,550 | 3,064 | 1,012 | 23 | 15 | 23 |
| 351611 | 1,433 | 1,574 | 837 | 153 | 95 | 168 |
| 351786 | 1,188 | 1,342 | 485 | 631 | 497 | 496 |
| 351829 | 459 | 464 | 258 | 1,479 | 1,168 | 1,205 |
| 352029 | 703 | 796 | 480 | 2,495 | 1,963 | 2,425 |
| 352272 | 1,734 | 1,236 | 1,600 | 14,660 | 13,524 | 7,157 |
| 352298 | 668 | 498 | 524 | 1,617 | 1,125 | 1,649 |
| 352535 | 862 | 887 | 525 | 1,602 | 1,205 | 1,424 |
| 352652 | 537 | 419 | 505 | 2,007 | 1,982 | 992 |
| 352811 | 555 | 494 | 449 | 1,413 | 1,229 | 901 |
| 353089 | 682 | 682 | 374 | 929 | 712 | 913 |
| 353495 | 185 | 129 | 154 | 312 | 278 | 189 |
| 353568 | 151 | 130 | 113 | 91 | 92 | 72 |
| 353639 | 883 | 745 | 629 | 4,946 | 4,544 | 3,239 |
| 343692 | 407 | 397 | 229 | 624 | 495 | 477 |
| 343701 | 681 | 560 | 551 | 3,159 | 3,112 | 1,382 |
| 343727 | 2,410 | 2,710 | 1,615 | 1,355 | 1,136 | 961 |
| 343753 | 941 | 891 | 579 | 1,837 | 1,427 | 1,594 |
| 343760 | 6,698 | 5,469 | 5,178 | 15,001 | 14,712 | 6,487 |
| 343761 | 476 | 546 | 301 | 1,207 | 971 | 973 |
| 343800 | 828 | 897 | 497 | 2,116 | 1,717 | 1,741 |
| 343827 | 1,258 | 1,363 | 609 | 22 | 15 | 23 |
| 343914 | 867 | 997 | 520 | 1,736 | 1,400 | 1,535 |
| 344161 | 910 | 876 | 609 | 1,378 | 1,133 | 1,110 |
| 344172 | 369 | 367 | 224 | 2,883 | 2,342 | 2,397 |
| 348629 | 1,409 | 1,629 | 923 | 520 | 408 | 535 |
| 348654 | 542 | 486 | 376 | 2,971 | 2,277 | 2,408 |
| 344327 | 1,346 | 1,350 | 822 | 763 | 647 | 618 |
| 344401 | 615 | 580 | 501 | 3,682 | 3,740 | 1,632 |
| 344427 | 520 | 435 | 407 | 287 | 248 | 198 |
| 344669 | 851 | 797 | 464 | 3,190 | 2,256 | 3,175 |
| 344720 | 728 | 641 | 531 | 1,498 | 1,124 | 1,454 |
| 344742 | 1,411 | 1,313 | 845 | 2,879 | 2,339 | 2,705 |
| 345131 | 1,952 | 1,794 | 1,340 | 6,410 | 6,010 | 4,018 |
| 345164 | 1,004 | 1,039 | 669 | 2,813 | 2,400 | 1,775 |
| 345254 | 284 | 262 | 150 | 1,282 | 1,014 | 1,077 |
| 345273 | 992 | 622 | 1,112 | 212 | 173 | 228 |
| 345302 | 641 | 565 | 520 | 1,509 | 1,353 | 1,075 |
| 345317 | 488 | 356 | 450 | 1,930 | 1,806 | 1,042 |
| 345327 | 1,259 | 1,231 | 832 | 437 | 350 | 425 |
| 345377 | 187 | 197 | 98 | 1,079 | 829 | 895 |
| 345400 | 586 | 558 | 372 | 1,767 | 1,441 | 1,382 |
| 345530 | 729 | 573 | 599 | 1,220 | 1,177 | 1,061 |
| 345564 | 687 | 750 | 402 | 3,981 | 3,076 | 3,272 |
| 345763 | 815 | 821 | 404 | 3,493 | 2,818 | 3,103 |
| 345802 | 1,962 | 1,255 | 2,180 | 31,152 | 28,182 | 17,806 |
| 345828 | 759 | 660 | 636 | 2,038 | 1,549 | 1,771 |
| 346065 | 332 | 296 | 221 | 1,112 | 907 | 871 |
| 346174 | 811 | 732 | 546 | 3,449 | 2,887 | 3,316 |
| 346243 | 2,099 | 1,954 | 1,174 | 861 | 735 | 654 |
| 346253 | 100 | 75 | 97 | 1,514 | 1,396 | 737 |
| 346384 | 6,106 | 4,142 | 6,427 | 829 | 568 | 898 |
| 346507 | 785 | 783 | 427 | 2,061 | 1,575 | 1,882 |
| 346541 | 564 | 560 | 382 | 1,203 | 764 | 1,426 |
| 346654 | 670 | 632 | 449 | 2,621 | 2,031 | 2,418 |
| 346711 | 732 | 703 | 437 | 1,898 | 1,394 | 1,896 |
| 346810 | 3,710 | 3,009 | 2,883 | 441 | 358 | 264 |
| 346877 | 2,713 | 2,471 | 1,890 | 6,913 | 6,544 | 4,282 |
| 346917 | 1,144 | 1,079 | 811 | 525 | 466 | 487 |
| 346918 | 541 | 563 | 312 | 146 | 94 | 135 |
| 346932 | 439 | 388 | 313 | 901 | 567 | 952 |
| 346983 | 185 | 168 | 110 | 46 | 36 | 45 |
| 346993 | 2,207 | 1,856 | 1,702 | 5,993 | 4,253 | 6,195 |
| 347144 | 2,348 | 2,180 | 1,536 | 803 | 606 | 707 |
| 347157 | 521 | 460 | 411 | 1,051 | 972 | 555 |
| 347205 | 1,605 | 1,598 | 1,049 | 3,297 | 2,898 | 2,288 |
| 347243 | 139 | 159 | 109 | 2,050 | 1,649 | 1,603 |
| 347355 | 216 | 202 | 199 | 1,723 | 1,569 | 951 |
| 347513 | 1,437 | 1,477 | 936 | 710 | 552 | 595 |
| 347518 | 1,276 | 1,074 | 789 | 2,977 | 2,525 | 2,078 |
| 347558 | 1,009 | 899 | 740 | 6,482 | 4,858 | 5,891 |
| 347569 | 636 | 627 | 511 | 1,710 | 1,570 | 1,028 |
| 347665 | 1,147 | 936 | 906 | 2,390 | 1,914 | 2,070 |
| 347713 | 2,936 | 1,292 | 3,470 | 84 | 79 | 83 |
| 347954 | 578 | 543 | 392 | 372 | 311 | 311 |
| 347958 | 1,345 | 1,573 | 924 | 2,797 | 2,569 | 2,261 |
| 347973 | 1,529 | 1,385 | 1,083 | 5,707 | 4,797 | 4,356 |
| 347989 | 1,295 | 1,061 | 891 | 3,050 | 2,887 | 1,724 |
| 348159 | 433 | 415 | 244 | 1,164 | 932 | 1,255 |
| 348330 | 2,452 | 2,891 | 900 | 349 | 126 | 635 |
| 348358 | 1,131 | 1,156 | 715 | 2,365 | 2,016 | 1,635 |
| 270410 | 77 | 69 | 56 | 424 | 385 | 262 |
| 270537 | 2,940 | 2,625 | 1,963 | 25,378 | 20,030 | 20,802 |
| 270614 | 1,004 | 1,093 | 619 | 2,111 | 1,659 | 1,629 |
| 270725 | 142 | 113 | 121 | 806 | 749 | 436 |
| 283607 | 793 | 743 | 666 | 3,263 | 3,393 | 1,606 |
| 270950 | 635 | 601 | 359 | 1,783 | 1,397 | 1,537 |
| 271180 | 245 | 272 | 130 | 1,191 | 900 | 1,030 |
| 271384 | 1,027 | 1,022 | 575 | 6,026 | 4,547 | 5,501 |
| 271396 | 1,617 | 1,687 | 882 | 2,695 | 1,878 | 2,873 |
| 271449 | 913 | 1,010 | 535 | 3,389 | 2,510 | 3,078 |
| 271753 | 746 | 834 | 438 | 1,821 | 1,388 | 1,646 |
| 272492 | 108 | 82 | 89 | 596 | 573 | 336 |
| 272535 | 672 | 586 | 487 | 2,162 | 1,661 | 1,922 |
| 272793 | 1,194 | 1,218 | 706 | 2,129 | 1,627 | 1,578 |
| 273225 | 1,157 | 935 | 859 | 3,083 | 2,715 | 2,141 |
| 273248 | 2,389 | 2,316 | 1,733 | 1,290 | 956 | 1,083 |
| 273274 | 524 | 469 | 297 | 2,418 | 2,382 | 1,515 |
| 273366 | 1,291 | 1,212 | 819 | 617 | 449 | 527 |
| 273593 | 1,569 | 1,482 | 1,226 | 226 | 191 | 215 |
| 273760 | 1,302 | 1,037 | 1,140 | 5,036 | 4,831 | 3,030 |
| 273933 | 1,595 | 1,723 | 871 | 2,693 | 2,217 | 2,095 |
| 274107 | 12,919 | 11,307 | 9,182 | 2,013 | 1,440 | 1,849 |
| 274386 | 9,158 | 8,680 | 5,715 | 36,709 | 28,917 | 31,935 |
| 274617 | 326 | 162 | 349 | 6,288 | 5,714 | 4,098 |
| 274686 | 1,036 | 1,083 | 631 | 2,741 | 2,174 | 2,195 |
| 274809 | 147 | 129 | 140 | 3,477 | 3,482 | 1,477 |
| 274896 | 639 | 632 | 421 | 2,372 | 2,107 | 1,543 |
| 275071 | 418 | 383 | 310 | 1,214 | 1,222 | 652 |
| 275222 | 1,442 | 1,556 | 958 | 617 | 474 | 572 |
| 276251 | 1,866 | 1,698 | 1,192 | 819 | 658 | 675 |
| 276287 | 2,072 | 1,998 | 1,348 | 632 | 450 | 629 |
| 276377 | 661 | 444 | 641 | 11,468 | 12,474 | 4,553 |
| 276480 | 1,090 | 1,257 | 657 | 99 | 87 | 95 |
| 276559 | 331 | 308 | 204 | 702 | 600 | 504 |
| 276622 | 4,329 | 3,933 | 3,168 | 1,628 | 1,175 | 1,586 |
| 276668 | 1,242 | 1,271 | 777 | 381 | 292 | 319 |
| 276762 | 530 | 483 | 363 | 1,102 | 843 | 985 |
| 277085 | 280 | 247 | 191 | 1,268 | 854 | 1,261 |
| 277466 | 73 | 56 | 77 | 5,052 | 5,201 | 1,792 |
| 277649 | 643 | 620 | 430 | 2,045 | 1,979 | 1,030 |
| 277862 | 36 | 40 | 41 | 1,242 | 1,299 | 543 |
| 278023 | 1,943 | 2,031 | 1,257 | 1,090 | 729 | 1,075 |
| 278137 | 106 | 107 | 67 | 179 | 135 | 184 |
| 278299 | 1,885 | 1,639 | 1,465 | 708 | 493 | 749 |
| 278354 | 3,811 | 3,482 | 2,897 | 6,592 | 6,044 | 3,818 |
| 278466 | 168 | 186 | 89 | 1,290 | 904 | 1,106 |
| 278980 | 261 | 209 | 195 | 3,470 | 3,355 | 2,013 |
| 279102 | 535 | 538 | 297 | 165 | 116 | 152 |
| 279251 | 13,348 | 15,289 | 7,605 | 2 | 4 | 1 |
| 279435 | 576 | 628 | 318 | 2,490 | 2,004 | 2,174 |
| 279861 | 697 | 747 | 379 | 3,138 | 2,403 | 2,768 |
| 279968 | 293 | 278 | 227 | 85 | 59 | 99 |
| 280546 | 2,527 | 2,431 | 1,686 | 1,029 | 815 | 811 |
| 280694 | 529 | 448 | 398 | 225 | 205 | 150 |
| 280920 | 1,315 | 1,360 | 840 | 2,556 | 2,181 | 1,943 |
| 281190 | 530 | 522 | 334 | 1,350 | 1,077 | 1,157 |
| 281441 | 749 | 533 | 711 | 1,856 | 1,610 | 1,429 |
| 281981 | 2,654 | 2,652 | 1,418 | 210 | 172 | 164 |
| 282324 | 813 | 731 | 677 | 1,749 | 1,472 | 1,571 |
| 282375 | 2,407 | 2,349 | 1,525 | 1,384 | 1,066 | 1,009 |
| 282721 | 499 | 528 | 327 | 1,400 | 1,208 | 1,021 |
| 283015 | 105 | 142 | 64 | 55 | 61 | 46 |
| 297116 | 3,570 | 3,266 | 2,486 | 2,157 | 1,413 | 1,951 |
| 283813 | 118 | 111 | 56 | 1,299 | 1,144 | 1,025 |
| 284045 | 502 | 541 | 307 | 1,223 | 963 | 867 |
| 284080 | 1,569 | 1,387 | 1,027 | 3,958 | 3,371 | 2,869 |
| 284134 | 2,560 | 2,592 | 1,676 | 1,208 | 999 | 985 |
| 284353 | 629 | 716 | 370 | 1,695 | 1,351 | 1,208 |
| 284422 | 1,680 | 1,715 | 1,048 | 3,886 | 3,233 | 2,960 |
| 284915 | 1,223 | 1,077 | 721 | 8,743 | 6,794 | 7,418 |
| 284950 | 4,146 | 4,361 | 2,243 | 745 | 523 | 765 |
| 285362 | 3,193 | 3,052 | 1,942 | 5,057 | 3,985 | 4,145 |
| 285367 | 1,281 | 1,296 | 727 | 137 | 124 | 101 |
| 285678 | 110 | 101 | 76 | 1,994 | 1,897 | 1,061 |
| 285725 | 1,193 | 1,110 | 782 | 3,538 | 2,947 | 2,365 |
| 285834 | 170 | 111 | 248 | 586 | 625 | 342 |
| 285853 | 3,473 | 2,514 | 3,513 | 404 | 368 | 451 |
| 285995 | 1,807 | 1,833 | 1,205 | 4,811 | 4,581 | 3,471 |
| 286042 | 520 | 492 | 378 | 1,058 | 923 | 841 |
| 286113 | 561 | 567 | 351 | 2,121 | 1,561 | 1,916 |
| 286241 | 1,329 | 1,198 | 715 | 266 | 221 | 316 |
| 286567 | 763 | 703 | 573 | 3,160 | 2,581 | 2,968 |
| 286719 | 955 | 806 | 794 | 3,235 | 2,943 | 1,779 |
| 286750 | 3,409 | 2,886 | 2,619 | 652 | 407 | 777 |
| 286788 | 577 | 579 | 445 | 2,491 | 2,303 | 2,546 |
| 286845 | 748 | 763 | 504 | 1,583 | 1,309 | 1,159 |
| 287030 | 537 | 542 | 295 | 2,772 | 2,392 | 2,286 |
| 287106 | 567 | 507 | 455 | 1,315 | 1,020 | 1,323 |
| 287346 | 609 | 497 | 345 | 1,132 | 1,002 | 768 |
| 287411 | 1,453 | 1,222 | 1,180 | 173 | 84 | 176 |
| 287419 | 547 | 614 | 320 | 4,125 | 3,843 | 2,840 |
| 287451 | 2,219 | 1,770 | 1,806 | 1,227 | 861 | 1,417 |
| 287461 | 2,251 | 2,045 | 1,751 | 1,518 | 1,482 | 1,035 |
| 287505 | 84 | 105 | 14 | 288 | 221 | 182 |
| 287530 | 1,751 | 1,304 | 1,673 | 5,062 | 5,048 | 2,069 |
| 287592 | 660 | 623 | 496 | 1,637 | 1,479 | 1,062 |
| 287614 | 511 | 344 | 601 | 5,755 | 5,517 | 2,424 |
| 287649 | 1,169 | 981 | 870 | 747 | 599 | 629 |
| 287728 | 1,481 | 1,356 | 981 | 3,881 | 3,218 | 2,626 |
| 287936 | 62 | 70 | 37 | 1,007 | 730 | 1,019 |
| 287977 | 2,837 | 2,609 | 2,123 | 768 | 672 | 644 |
| 288210 | 3,207 | 2,736 | 2,243 | 958 | 818 | 865 |
| 288281 | 864 | 812 | 642 | 370 | 282 | 298 |
| 288460 | 1,576 | 1,562 | 1,017 | 3,529 | 2,623 | 2,573 |
| 288798 | 256 | 214 | 154 | 1,105 | 838 | 992 |
| 288810 | 4,148 | 4,172 | 2,502 | 7,838 | 7,215 | 6,041 |
| 288852 | 1,351 | 1,365 | 854 | 4,057 | 3,426 | 2,868 |
| 288955 | 1,341 | 1,176 | 785 | 732 | 557 | 684 |
| 288979 | 1,260 | 1,284 | 768 | 268 | 209 | 213 |
| 289501 | 1,452 | 1,103 | 1,447 | 22,298 | 21,145 | 11,218 |
| 289601 | 384 | 352 | 329 | 1,323 | 1,347 | 654 |
| 289645 | 1,419 | 1,555 | 793 | 438 | 344 | 452 |
| 289793 | 1,151 | 1,034 | 944 | 3,502 | 2,704 | 2,787 |
| 289896 | 709 | 652 | 520 | 1,523 | 1,312 | 1,094 |
| 290023 | 3,602 | 3,076 | 2,631 | 5,249 | 4,212 | 4,652 |
| 290351 | 1,151 | 1,034 | 726 | 3,451 | 2,707 | 2,911 |
| 290551 | 258 | 238 | 208 | 902 | 895 | 508 |
| 290625 | 3,181 | 3,129 | 2,088 | 1,045 | 820 | 997 |
| 290756 | 2,394 | 1,680 | 2,004 | 268 | 140 | 401 |
| 291319 | 979 | 994 | 683 | 2,901 | 2,490 | 2,226 |
| 291478 | 617 | 566 | 395 | 1,962 | 1,497 | 1,976 |
| 291759 | 785 | 690 | 571 | 1,716 | 1,382 | 1,163 |
| 291779 | 203 | 106 | 213 | 5,231 | 5,317 | 2,166 |
| 291806 | 4,617 | 4,136 | 3,344 | 9,672 | 7,613 | 8,619 |
| 291836 | 2,366 | 2,507 | 1,281 | 9,767 | 7,536 | 8,104 |
| 292001 | 454 | 465 | 269 | 2,102 | 1,577 | 1,693 |
| 292243 | 2,819 | 2,585 | 2,002 | 6,303 | 4,426 | 6,026 |
| 292291 | 1,189 | 1,167 | 658 | 624 | 402 | 559 |
| 292381 | 947 | 754 | 774 | 402 | 333 | 446 |
| 292479 | 1,185 | 1,055 | 741 | 376 | 287 | 305 |
| 292545 | 705 | 637 | 426 | 1,440 | 1,112 | 1,172 |
| 292762 | 716 | 678 | 437 | 1,102 | 893 | 855 |
| 292843 | 27,572 | 28,705 | 13,959 | 146 | 89 | 94 |
| 293185 | 800 | 780 | 510 | 1,694 | 1,416 | 1,211 |
| 293581 | 1,248 | 1,007 | 1,153 | 8,637 | 7,130 | 7,395 |
| 293608 | 1,173 | 895 | 889 | 7,317 | 5,454 | 5,994 |
| 293774 | 1,762 | 1,955 | 829 | 225 | 171 | 221 |
| 293944 | 243 | 260 | 158 | 991 | 866 | 694 |
| 294047 | 948 | 969 | 579 | 413 | 301 | 294 |
| 294188 | 323 | 295 | 175 | 2,086 | 1,520 | 1,796 |
| 294443 | 612 | 636 | 317 | 1,300 | 973 | 961 |
| 294560 | 1,336 | 1,188 | 979 | 2,638 | 2,105 | 2,283 |
| 294704 | 288 | 210 | 209 | 869 | 699 | 606 |
| 294870 | 1,545 | 1,498 | 1,002 | 2,450 | 2,132 | 2,086 |
| 294973 | 445 | 461 | 263 | 2,886 | 2,349 | 2,177 |
| 295194 | 1,028 | 1,000 | 742 | 445 | 328 | 416 |
| 295293 | 1,589 | 1,166 | 1,553 | 5,682 | 6,276 | 2,499 |
| 295661 | 184 | 163 | 180 | 1,336 | 928 | 1,573 |
| 295721 | 66 | 80 | 51 | 1,954 | 1,854 | 1,150 |
| 295967 | 411 | 439 | 249 | 1,084 | 782 | 938 |
| 296367 | 1,884 | 1,882 | 1,247 | 340 | 245 | 260 |
| 296388 | 48 | 31 | 27 | 1,707 | 1,775 | 734 |
| 296428 | 422 | 391 | 330 | 64 | 50 | 62 |
| 296580 | 774 | 778 | 589 | 288 | 240 | 319 |
| 296739 | 60 | 65 | 48 | 1,190 | 1,099 | 601 |
| 320020 | 2,467 | 2,375 | 1,561 | 559 | 423 | 432 |
| 320079 | 93 | 136 | 61 | 188 | 186 | 129 |
| 320080 | 253 | 294 | 133 | 471 | 374 | 319 |
| 320360 | 2,366 | 2,767 | 1,521 | 5,262 | 4,097 | 4,283 |
| 320361 | 2,870 | 2,613 | 2,225 | 4,923 | 4,360 | 3,217 |
| 320448 | 14,158 | 16,292 | 8,920 | 38,373 | 33,154 | 26,228 |
| 320451 | 20,088 | 25,851 | 10,883 | 63,081 | 53,463 | 36,729 |
| 320486 | 1,351 | 1,582 | 830 | 2,862 | 2,382 | 2,433 |
| 320529 | 2,025 | 1,800 | 1,318 | 6,465 | 5,173 | 5,875 |
| 320691 | 411 | 389 | 335 | 2,649 | 2,785 | 1,310 |
| 320764 | 1,926 | 1,831 | 1,331 | 409 | 305 | 439 |
| 320811 | 6,682 | 8,514 | 3,480 | 21,388 | 17,988 | 12,014 |
| 324686 | 10,697 | 13,731 | 5,274 | 33,406 | 27,611 | 18,122 |
| 324677 | 20,087 | 25,851 | 10,883 | 63,081 | 53,462 | 36,729 |
| 324681 | 14,712 | 16,931 | 9,223 | 39,982 | 34,572 | 27,283 |
| 324763 | 5,826 | 7,475 | 2,924 | 18,160 | 14,921 | 10,005 |
| 324822 | 22,739 | 26,135 | 14,543 | 60,547 | 52,460 | 42,637 |
| 324836 | 7,979 | 10,338 | 4,114 | 24,540 | 20,614 | 13,856 |
| 324852 | 529 | 574 | 367 | 1,539 | 1,356 | 1,252 |
| 324826 | 22,369 | 28,835 | 12,078 | 69,795 | 59,056 | 40,517 |
| 324911 | 10,474 | 12,247 | 6,582 | 28,636 | 24,987 | 19,590 |
| 324913 | 4,138 | 5,402 | 2,173 | 13,238 | 11,082 | 7,378 |
| 324918 | 20,099 | 23,131 | 12,869 | 53,545 | 46,344 | 37,589 |
| 324921 | 4,872 | 6,234 | 2,447 | 15,257 | 12,686 | 8,351 |
| 324924 | 21,727 | 28,112 | 11,794 | 68,128 | 58,015 | 39,857 |
| 320973 | 743 | 774 | 545 | 1,712 | 1,473 | 1,435 |
| 320974 | 362 | 431 | 273 | 880 | 859 | 879 |
| 320831 | 10,524 | 12,135 | 6,658 | 28,319 | 24,594 | 19,433 |
| 320832 | 20,180 | 26,023 | 10,764 | 63,260 | 53,273 | 36,416 |
| 321049 | 311 | 365 | 119 | 2,650 | 1,832 | 3,160 |
| 320837 | 1,248 | 1,292 | 815 | 2,624 | 2,105 | 3,290 |
| 321126 | 712 | 580 | 645 | 1,922 | 1,733 | 1,531 |
| 321141 | 597 | 547 | 500 | 1,464 | 1,171 | 1,296 |
| 321207 | 959 | 1,183 | 342 | 3,211 | 2,373 | 1,349 |
| 321241 | 1,181 | 1,152 | 813 | 273 | 227 | 200 |
| 320855 | 22,113 | 28,490 | 11,901 | 68,728 | 57,909 | 39,736 |
| 321384 | 108 | 77 | 80 | 1,632 | 1,118 | 1,685 |
| 321284 | 3,973 | 3,278 | 3,495 | 17,420 | 17,264 | 8,268 |
| 321518 | 1,628 | 1,745 | 1,017 | 318 | 215 | 355 |
| 321522 | 1,461 | 1,181 | 996 | 855 | 553 | 855 |
| 321525 | 143 | 146 | 121 | 548 | 475 | 333 |
| 321733 | 969 | 870 | 702 | 2,794 | 2,360 | 2,053 |
| 321783 | 1,045 | 996 | 751 | 451 | 327 | 399 |
| 321786 | 2,233 | 2,470 | 1,566 | 1,364 | 1,191 | 937 |
| 321800 | 172 | 153 | 181 | 861 | 812 | 411 |
| 321341 | 22,389 | 28,985 | 11,561 | 69,021 | 57,837 | 39,141 |
| 322001 | 648 | 575 | 501 | 1,100 | 1,037 | 718 |
| 321882 | 186 | 135 | 159 | 3,998 | 3,905 | 1,854 |
| 322146 | 415 | 479 | 216 | 1,188 | 1,050 | 903 |
| 322154 | 54,120 | 69,999 | 27,662 | 167,628 | 139,604 | 92,447 |
| 322273 | 989 | 945 | 613 | 1,752 | 1,399 | 1,364 |
| 321903 | 1,812 | 1,877 | 1,264 | 9,813 | 7,930 | 9,302 |
| 322307 | 5,921 | 6,404 | 3,607 | 3,730 | 2,979 | 3,575 |
| 322323 | 853 | 942 | 595 | 331 | 283 | 262 |
| 321913 | 829 | 912 | 565 | 6,036 | 4,699 | 5,940 |
| 321922 | 2,287 | 2,889 | 1,107 | 7,529 | 6,250 | 3,952 |
| 321925 | 30,984 | 40,099 | 15,875 | 96,886 | 80,836 | 53,585 |
| 322454 | 25,211 | 28,923 | 16,136 | 66,996 | 58,188 | 47,561 |
| 322584 | 852 | 848 | 631 | 365 | 309 | 243 |
| 322594 | 22,644 | 25,718 | 14,505 | 60,086 | 52,079 | 42,710 |
| 322595 | 11,354 | 14,666 | 5,551 | 35,821 | 29,670 | 19,280 |
| 322606 | 884 | 1,037 | 496 | 10,859 | 7,749 | 10,037 |
| 322631 | 1,140 | 1,207 | 667 | 141 | 107 | 146 |
| 322637 | 1,902 | 1,988 | 1,047 | 5,322 | 4,279 | 4,430 |
| 322655 | 7,933 | 10,206 | 3,896 | 24,786 | 20,372 | 13,365 |
| 322716 | 1,163 | 980 | 962 | 4,034 | 3,854 | 3,040 |
| 322738 | 1,211 | 1,260 | 863 | 531 | 432 | 435 |
| 322850 | 932 | 865 | 707 | 2,028 | 1,564 | 1,750 |
| 322863 | 1,069 | 1,105 | 718 | 12 | 9 | 13 |
| 322929 | 2,859 | 2,152 | 2,252 | 4,518 | 3,078 | 4,755 |
| 322968 | 20,068 | 25,829 | 10,878 | 63,011 | 53,411 | 36,689 |
| 323091 | 252 | 222 | 180 | 1,854 | 1,355 | 1,935 |
| 323138 | 1,284 | 1,161 | 907 | 472 | 377 | 574 |
| 323187 | 5,628 | 7,217 | 2,732 | 17,955 | 14,777 | 9,713 |
| 323268 | 39,583 | 51,077 | 20,254 | 122,141 | 101,905 | 67,881 |
| 323047 | 2,537 | 2,899 | 1,408 | 4 | 6 | 5 |
| 323049 | 3,384 | 3,706 | 1,911 | 2 | 1 | 3 |
| 323583 | 7,042 | 6,904 | 4,615 | 3,356 | 2,579 | 2,572 |
| 323593 | 1,041 | 1,020 | 656 | 4,688 | 3,546 | 3,941 |
| 323670 | 2,215 | 1,322 | 2,552 | 7,957 | 8,306 | 3,013 |
| 323446 | 1,370 | 1,330 | 1,049 | 3,820 | 2,928 | 2,834 |
| 323704 | 1,140 | 1,224 | 706 | 23 | 29 | 11 |
| 323706 | 131 | 165 | 63 | 477 | 404 | 346 |
| 323712 | 24,058 | 31,463 | 13,491 | 72,803 | 62,258 | 43,804 |
| 323714 | 22,424 | 28,937 | 12,101 | 70,134 | 59,365 | 40,591 |
| 323727 | 3,142 | 2,549 | 2,564 | 5,550 | 4,285 | 5,627 |
| 323764 | 51,190 | 66,113 | 26,121 | 158,226 | 131,830 | 87,427 |
| 323765 | 25,297 | 29,023 | 16,202 | 67,307 | 58,414 | 47,776 |
| 323494 | 472 | 488 | 352 | 289 | 236 | 179 |
| 323766 | 21,872 | 25,006 | 13,968 | 58,367 | 50,473 | 41,392 |
| 323868 | 1,247 | 1,087 | 1,115 | 2,501 | 2,168 | 1,873 |
| 323872 | 1,858 | 1,897 | 1,279 | 851 | 727 | 651 |
| 323768 | 28,265 | 36,474 | 14,406 | 88,063 | 73,451 | 48,908 |
| 324291 | 580 | 522 | 453 | 1,337 | 1,010 | 1,201 |
| 323819 | 15,307 | 19,638 | 7,836 | 47,166 | 39,339 | 26,402 |
| 323774 | 12,863 | 16,453 | 6,395 | 39,605 | 32,708 | 21,568 |
| 324608 | 804 | 534 | 948 | 1,493 | 1,239 | 1,436 |
| 324653 | 733 | 652 | 479 | 1,306 | 1,087 | 1,282 |
| 324353 | 1,023 | 742 | 1,069 | 6,842 | 5,660 | 5,895 |
| 324322 | 4,100 | 5,158 | 2,031 | 12,470 | 10,263 | 6,963 |
| 326095 | 375 | 350 | 209 | 1,130 | 996 | 790 |
| 326345 | 811 | 874 | 525 | 1,717 | 1,418 | 1,535 |
| 325080 | 447 | 505 | 294 | 4,220 | 3,057 | 3,754 |
| 324947 | 38 | 31 | 28 | 234 | 179 | 128 |
| 325579 | 381 | 459 | 223 | 1,814 | 1,306 | 1,519 |
| 325835 | 433 | 406 | 229 | 1,066 | 825 | 777 |
| 334808 | 266 | 268 | 121 | 2,240 | 1,609 | 1,953 |
| 335052 | 1,312 | 1,618 | 526 | 10 | 9 | 5 |
| 328810 | 853 | 864 | 587 | 1,425 | 1,237 | 1,013 |
| 329053 | 1,618 | 1,550 | 854 | 4,463 | 3,451 | 3,773 |
| 328135 | 1,856 | 1,427 | 1,549 | 632 | 494 | 612 |
| 328205 | 655 | 667 | 351 | 1,352 | 1,155 | 1,187 |
| 327767 | 314 | 208 | 254 | 516 | 518 | 207 |
| 328460 | 605 | 529 | 347 | 1,582 | 1,297 | 1,542 |
| 328511 | 625 | 607 | 434 | 1,358 | 1,072 | 1,155 |
| 328619 | 176 | 189 | 117 | 977 | 802 | 1,084 |
| 328700 | 487 | 432 | 442 | 1,549 | 1,524 | 1,023 |
| 329298 | 266 | 268 | 121 | 2,240 | 1,609 | 1,953 |
| 330344 | 193 | 212 | 118 | 60 | 60 | 54 |
| 329199 | 303 | 271 | 274 | 3,148 | 2,813 | 1,739 |
| 330529 | 414 | 413 | 252 | 1,741 | 1,404 | 1,658 |
| 330619 | 1,207 | 1,105 | 726 | 444 | 343 | 310 |
| 330780 | 1,364 | 917 | 1,399 | 6,130 | 5,473 | 4,755 |
| 330996 | 1,717 | 1,312 | 1,645 | 843 | 578 | 789 |
| 331026 | 2,006 | 1,848 | 1,351 | 12,383 | 10,896 | 8,487 |
| 331403 | 127 | 166 | 94 | 509 | 453 | 338 |
| 332007 | 1,241 | 821 | 1,267 | 5,513 | 4,941 | 4,307 |
| 332140 | 565 | 582 | 408 | 864 | 669 | 842 |
| 332170 | 266 | 268 | 121 | 2,240 | 1,609 | 1,953 |
| 332492 | 558 | 506 | 317 | 316 | 253 | 254 |
| 333204 | 1,241 | 821 | 1,267 | 5,513 | 4,941 | 4,307 |
| 333251 | 1,205 | 897 | 1,159 | 5,277 | 5,361 | 2,339 |
| 333461 | 215 | 175 | 150 | 1,960 | 1,939 | 997 |
| 333721 | 1,578 | 1,356 | 1,018 | 3,034 | 2,669 | 2,165 |
| 333952 | 79 | 72 | 57 | 7,992 | 8,105 | 3,284 |
| 334267 | 129 | 87 | 79 | 1,494 | 1,257 | 1,462 |
| 334312 | 266 | 268 | 121 | 2,240 | 1,609 | 1,953 |
| 334470 | 809 | 692 | 600 | 1,940 | 1,833 | 1,318 |
| 334807 | 266 | 268 | 121 | 2,240 | 1,609 | 1,953 |
| 336360 | 259 | 273 | 99 | 1,888 | 1,586 | 1,648 |
| 335419 | 519 | 428 | 385 | 2,557 | 2,441 | 1,526 |
| 336418 | 339 | 343 | 197 | 573 | 488 | 432 |
| 336441 | 570 | 569 | 322 | 2,152 | 1,606 | 1,998 |
| 336493 | 413 | 437 | 237 | 1,072 | 880 | 939 |
| 336556 | 268 | 245 | 174 | 1,222 | 1,030 | 829 |
| 336567 | 629 | 536 | 409 | 224 | 193 | 174 |
| 335584 | 309 | 252 | 187 | 471 | 320 | 526 |
| 335903 | 148 | 148 | 94 | 1,114 | 913 | 817 |
| 335957 | 633 | 645 | 338 | 3,021 | 2,250 | 2,750 |
| 336213 | 1,655 | 1,704 | 1,115 | 825 | 638 | 713 |
| 336228 | 179 | 211 | 113 | 1,287 | 948 | 1,137 |
| 343440 | 487 | 536 | 315 | 138 | 121 | 101 |
| 343514 | 3 | 1 | 2 | 75 | 68 | 63 |
| 343643 | 1,271 | 1,199 | 871 | 225 | 192 | 191 |
| 343596 | 1,622 | 1,313 | 1,111 | 271 | 204 | 246 |
| 337118 | 476 | 391 | 388 | 1,141 | 1,086 | 782 |
| 337221 | 408 | 349 | 281 | 1,526 | 1,140 | 1,317 |
| 337277 | 822 | 874 | 623 | 3,571 | 2,570 | 3,717 |
| 337516 | 728 | 664 | 501 | 366 | 255 | 296 |
| 337744 | 677 | 499 | 536 | 1,441 | 843 | 1,781 |
| 337763 | 458 | 469 | 245 | 2,602 | 1,906 | 2,317 |
| 337622 | 1,840 | 1,500 | 1,467 | 4,107 | 3,785 | 2,881 |
| 337606 | 2,750 | 1,889 | 2,901 | 11,073 | 10,593 | 5,864 |
| 337866 | 130 | 134 | 59 | 2,559 | 1,787 | 2,407 |
| 338281 | 207 | 267 | 102 | 1,075 | 912 | 895 |
| 338319 | 3,975 | 3,933 | 2,253 | 9,047 | 6,987 | 7,509 |
| 338561 | 236 | 233 | 148 | 1,125 | 808 | 1,104 |
| 338794 | 2,071 | 1,284 | 1,989 | 19,092 | 17,832 | 11,433 |
| 339052 | 76 | 55 | 59 | 342 | 302 | 153 |
| 338832 | 64 | 64 | 27 | 585 | 476 | 264 |
| 339678 | 387 | 319 | 374 | 2,120 | 1,764 | 1,390 |
| 339736 | 511 | 483 | 346 | 2,095 | 1,836 | 1,482 |
| 339743 | 95 | 82 | 58 | 1,435 | 996 | 1,455 |
| 340018 | 194 | 236 | 130 | 1,186 | 905 | 985 |
| 340321 | 261 | 256 | 116 | 1,303 | 935 | 1,118 |
| 340375 | 941 | 870 | 605 | 2,231 | 1,593 | 2,004 |
| 340658 | 408 | 422 | 302 | 1,061 | 952 | 620 |
| 340800 | 602,266 | 502,991 | 504,425 | 273,738 | 299,799 | 162,734 |
| 340992 | 1,928 | 1,711 | 1,357 | 38 | 39 | 16 |
| 341496 | 388 | 286 | 316 | 7,084 | 7,013 | 3,263 |
| 341546 | 1,823 | 1,692 | 1,278 | 4,116 | 3,271 | 3,092 |
| 341914 | 1,438 | 1,406 | 775 | 2,780 | 2,095 | 2,657 |
| 341946 | 1,183 | 999 | 862 | 576 | 424 | 468 |
| 342213 | 1,102 | 1,100 | 645 | 3,354 | 2,557 | 3,193 |
| 342243 | 81 | 86 | 25 | 530 | 391 | 381 |
| 342244 | 333 | 335 | 124 | 1,769 | 1,449 | 1,395 |
| 342285 | 656 | 727 | 324 | 1,417 | 1,023 | 1,502 |
| 342304 | 422 | 434 | 291 | 916 | 781 | 690 |
| 343078 | 671 | 627 | 419 | 967 | 616 | 1,097 |
| 343250 | 311 | 250 | 203 | 1,890 | 1,864 | 955 |
| 225273 | 160 | 188 | 71 | 1,415 | 1,150 | 1,007 |
| 241669 | 507 | 539 | 293 | 1,694 | 1,242 | 1,442 |
| 229564 | 445 | 317 | 383 | 1,704 | 1,380 | 1,255 |
| 233964 | 87 | 64 | 56 | 303 | 261 | 174 |
| 231598 | 305 | 247 | 253 | 1,172 | 1,018 | 650 |
| 227798 | 551 | 558 | 365 | 1,238 | 912 | 1,045 |
| 236899 | 302 | 315 | 145 | 1,494 | 1,186 | 1,257 |
| 227134 | 2,965 | 2,395 | 2,242 | 5,199 | 4,352 | 3,929 |
| 233499 | 717 | 532 | 773 | 4,464 | 4,761 | 1,756 |
| 236834 | 316 | 314 | 181 | 1,620 | 1,288 | 1,172 |
| 218589 | 922 | 899 | 572 | 1,840 | 1,578 | 1,422 |
| 217557 | 429 | 417 | 271 | 1,261 | 920 | 988 |
| 244454 | 1,108 | 1,085 | 748 | 686 | 664 | 284 |
| 242106 | 1,394 | 1,397 | 680 | 421 | 349 | 410 |
| 239131 | 273 | 286 | 194 | 106 | 61 | 87 |
| 242884 | 3,092 | 3,072 | 1,598 | 654 | 449 | 572 |
| 220966 | 13 | 32 | 1 | 1,357 | 1,413 | 530 |
| 226732 | 859 | 812 | 476 | 3,136 | 2,355 | 2,598 |
| 216035 | 1,006 | 952 | 516 | 4,895 | 3,733 | 4,264 |
