# Supplementary material for: Transcriptome Analysis of Cryphonectria parasitica Infected With Cryphonectria hypovirus 1 (CHV1) Reveals Distinct Genes Related to Fungal Metabolites, Virulence, Antiviral RNA-Silencing, and Their Regulation
Source: Front Microbiol. 2020 Jul 17;11:1711. doi: 10.3389/fmicb.2020.01711 (PMC7379330; doi:10.3389/fmicb.2020.01711)
Supplement: Supplementary file 3 [file Table_2.DOCX]

**Table S2 |** Sequences of the primer pairs used in the real-time RT-PCR

| JGI gene ID |  | Primer | Fragment size |
| --- | --- | --- | --- |
| 85578 | Forward  Reverse | 5’-CCACATCTGAAAAACCCTCAA-3’  5’-CCGAGGGGTAGCCTCTTTAC-3’ | 178 |
| 356022 | Forward  Reverse | 5’-TACTTTGGCATGGACAACGA-3’  5’-TGTAGAGAACGGGAGGGTTG-3 | 202 |
| 67838 | Forward  Reverse | 5’-GGATCAGATATGGCGCTTGT-3’  5’-CAAAGGAAAGCTTGGTCTGC-3 | 160 |
| 330996 | Forward  Reverse | 5’-GAAATTTGCCTTTTCGGTCA-3’  5’-TTGGGTCCGAAAGATAGACG-3 | 221 |
| 355825 | Forward  Reverse | 5’-AGCCTGACAAGACTGCTGGT-3’  5’-GCTGGCAGAGGGTACTCAAG-3 | 244 |
| 348629 | Forward  Reverse | 5’-GTGACCATGTGCCTCCTCCT-3’  5’-CATAATTCTGCCCCTGGTTC-3 | 180 |
| 109204 | Forward  Reverse | 5’-CATGTCGGATACAGACCTCA-3’  5’-TTTTTCCTGGTTGTCGCTTC-3 | 190 |
| 358511 | Forward  Reverse | 5’-CTGGGATCATCTCCAAGCAT-3’  5’-GCTTGATCCAAGTCGAGAGG-3 | 191 |
| 248975 | Forward  Reverse | 5’-CCGGTGAGGGTGACTACTGT-3’  5’-TCTTGCAGCTGGTTGTTGAG-3 | 168 |
| 284134 | Forward  Reverse | 5’-GCGCACATACTGGTACCTCA-3’  5’-GGCACGTATAGCTGGGGATA-3 | 207 |
| 357263 | Forward  Reverse | 5’-GACAAGGTCAGCGTCTCCTC-3’  5’-GGTACGTAGGAGTGCGTGGT-3 | 161 |
| 287030 | Forward  Reverse | 5’-CACAAGAAGGACGCACTCAA-3’  5’-AATATCATTGCCCACCGAAA-3 | 153 |
| 356265 | Forward  Reverse | 5’-CAGGAGGATCTGCAGAGGAG-3’  5’-GCCCTGATTCACGTTCTTGT-3 | 165 |
| 225273 | Forward  Reverse | 5’-CTGGTCAGGAGTGTGCAAGA-3’  5’-TGACGCGTACTAGCTTGTGG-3 | 160 |
| 105038 | Forward  Reverse | 5’-GACACGCACTTGACCTCTGA-3’  5’-TGAACTACACGCTCCGGAAT-3 | 164 |
| 104198 | Forward  Reverse | 5’-GCTTGCAGCTCCACTCTGTA-3’  5’-AGAGGAATCGTGCAGCACTT-3 | 167 |
| 64984 | Forward  Reverse | 5’-TCACACTTTGGGGCCTCTAC-3’  5’-GTGTTGAAAGGGCCAAACC-3 | 175 |
| 32824 | Forward  Reverse | 5’-ATCTCTGGCAAGGACACTGG-3’  5’-TCCCTGGATCTGTCCAAAAG-3 | 172 |
| 39251 | Forward  Reverse | 5’-CGTCCCTATCGTCCCTTACA-3’  5’-TCACGTTGCTAGTGCTGGTC-3 | 215 |
| 358133 | Forward  Reverse | 5-GACGGCATCATCGACATC-3’  5’-GGGTGTAGGCACAACAATGG-3 | 225 |
